# Evaluation of satellite and reanalysis‐based global net surface energy flux and uncertainty estimates

**DOI:** 10.1002/2017JD026616

**Published:** 2017-06-26

**Authors:** Chunlei Liu, Richard P. Allan, Michael Mayer, Patrick Hyder, Norman G. Loeb, Chris D. Roberts, Maria Valdivieso, John M. Edwards, Pier‐Luigi Vidale

**Affiliations:** ^1^ Department of Meteorology University of Reading Reading UK; ^2^ National Centre for Earth Observation Reading UK; ^3^ National Centre for Atmospheric Science Reading UK; ^4^ Department of Meteorology and Geophysics University of Vienna Vienna Austria; ^5^ Met Office Exeter UK; ^6^ NASA Langley Research Centre Hampton Virginia USA

**Keywords:** net surface energy flux, mass correction, land surface flux constraint, uncertainty, interhemispheric energy imbalance

## Abstract

The net surface energy flux is central to the climate system yet observational limitations lead to substantial uncertainty. A combination of satellite‐derived radiative fluxes at the top of atmosphere adjusted using the latest estimation of the net heat uptake of the Earth system, and the atmospheric energy tendencies and transports from the ERA‐Interim reanalysis are used to estimate surface energy flux globally. To consider snowmelt and improve regional realism, land surface fluxes are adjusted through a simple energy balance approach at each grid point. This energy adjustment is redistributed over the oceans to ensure energy conservation and maintain realistic global ocean heat uptake, using a weighting function to avoid meridional discontinuities. Calculated surface energy fluxes are evaluated through comparison to ocean reanalyses. Derived turbulent energy flux variability is compared with the Objectively Analyzed air‐sea Fluxes (OAFLUX) product, and inferred meridional energy transports in the global ocean and the Atlantic are also evaluated using observations. Uncertainties in surface fluxes are investigated using a variety of approaches including comparison with a range of atmospheric reanalysis products. Decadal changes in the global mean and the interhemispheric energy imbalances are quantified, and present day cross‐equator heat transports are reevaluated at 0.22 ± 0.15 PW (petawatts) southward by the atmosphere and 0.32 ± 0.16 PW northward by the ocean considering the observed ocean heat sinks.

## Key Points

- Improved global net surface energy flux estimates by combining satellite and atmospheric reanalysis data
- Zonal multiannual mean standard deviation across five reanalysis surface flux estimates (11 Wm^−2^) reduced to 2.6 Wm^−2^ using new method
- Cross‐equator heat transport reevaluated: 0.22 PW to south by atmosphere and 0.32 PW to north by ocean

## 1 Introduction

The net surface flux (*F*_*s*_ ) plays a key role in determining the decadal surface temperature variability [*Easterling and Wehner*, 2009; *Knight et al*., 2009; *Trenberth and Fasullo*, 2013; *Huber and Knutti*, 2014; *Watanabe et al*., 2014]. The incoming short‐wave radiation provides much of the energy required for surface water evaporation and modulates the surface temperature. The net downward surface energy can accumulate within the ocean, driving long‐term climate change [*Otto et al*., 2013; *Richardson et al*., 2016]. Since observations of surface fluxes are sparse [e.g., *Wild et al*., 2015], radiative transfer models are commonly used with observed atmospheric meteorological parameters as input, such as satellite‐derived cloud properties, to compute radiative fluxes. Turbulent heat fluxes are typically approximated by bulk formulae using surface variables, such as the observed wind and temperature [*Schmetz*, 1991; *Singh et al*., 2005]. Since homogeneity and accuracy of computed fluxes are questionable [*Mayer et al*., 2013], an alternative approach is to combine satellite observations of top of atmosphere radiative fluxes with atmospheric energy transports from reanalyses to estimate the global net surface energy fluxes as a residual [*Trenberth and Solomon*, 1994]. Applying this strategy, *Liu et al*. [2015] found that the divergence of horizontal atmospheric energy transports (hereafter: energy divergences) over land required adjustments to remove unrealistically large implied surface energy fluxes over land based upon simple energy budget arguments. Estimates of atmospheric energy transport vary greatly across the reanalysis systems due to the large and varying impacts of changes in the observing system [*Trenberth and Fasullo*, 2013]. The necessary adjustments may reduce the utility of the land surface fluxes, but the primary goal of the data set is to generate realistic surface fluxes over oceans, which is more crucial because it influences ocean heat uptake and transport as well as regional climate feedbacks [*Brown et al*., 2016]. The resulting surface data set has already been used by other investigators [*Williams et al*., 2015; *Valdivieso et al*., 2015; *Senior et al*., 2016; *Roberts et al*., 2017] for surface flux comparisons and model simulation evaluations, yet a lack of information regarding uncertainty estimates currently limits its value. In the present study, an extended, improved estimate of the top of atmosphere radiation budget with reduced absolute uncertainty is applied and a modified method is used to constrain land surface fluxes. The estimated surface flux variabilities are compared with those from ocean reanalyses, including GODAS (Global Ocean Data Assimilation System) [*Behringer*, 2007], C‐GLORS05v3 (CMCC Global Ocean Physical Reanalysis System) [*Storto et al*., 2014], and ORAS4 (Ocean Reanalysis System 4) [*Balmaseda et al*., 2013], for selected regions. The inferred meridional oceanic energy transports are compared with observations in the global ocean and the Atlantic. The derived turbulent energy flux variabilities using additional satellite‐based surface radiative flux estimates are also compared with those from the OAFLUX (Objectively Analyzed air‐sea Fluxes) [*Yu et al*., 2008] product for a number of regions. The composite observations from buoy stations of TAO/TRITON (Tropical Atmosphere Ocean/Triangle Trans‐Ocean Buoy Network) [*McPhaden et al*., 1998], RAMA (Research Moored Array for African‐Asian‐Australian Monsoon Analysis and Prediction) [*McPhaden et al*., 2009], and PIRATA (Prediction and Research Moored Array in the Atlantic) [*Bourles et al*., 2008] are also used for turbulent flux comparisons in six regions. The uncertainty in surface fluxes is then estimated using a variety of approaches. The variability in the regional energy fluxes, updated estimates of interhemispheric energy imbalances, and cross‐equatorial heat transports are provided using the new reconstructed data and accounting for the observed patterns of oceanic heat accumulation.

## 2 Data and Methods

### 2.1 General Approach

Surface downward energy flux (*F*_*s*_ ) is calculated at each grid point for each month by combining satellite‐based top‐of‐atmosphere (TOA) net radiation (*F*_*T*_ ) with column integrated atmospheric horizontal energy divergences and tendencies (changes in energy storage within the atmospheric column) and ensuring energy conservation:

(1) $$F_{S}=F_{T}-\frac{\partial E}{\partial t}-\nabla \cdot \frac{1}{g}\int_{0}^{1}V\left(h+k\right)\frac{\partial p}{\partial \eta }d\eta$$

where *E* is the total atmospheric energy. The total energy tendency, $\frac{\text{∂}E}{\text{∂}t}$, is small compared with other terms and can be calculated from time series of *E* computed from reanalyses. *g* is the gravitational acceleration, ***V*** is the horizontal wind velocity vector, *h* is the moist static energy, and *k* is the kinetic energy. *p* is the pressure and *η* is the hybrid vertical coordinate which is a function of atmospheric and surface pressure [*Simmons and Burridge*, 1981]. $\text{∇}\cdot \frac{1}{g}\int_{0}^{1}V\left(h+k\right)\;\frac{\text{∂}p}{\text{∂}\eta }d\eta$ is the divergence of vertically integrated horizontal energy transport. For mass consistency, the atmospheric energy divergence from ERA‐Interim reanalysis is mass corrected [*Trenberth et al*., 1995; *Chiodo and Haimberger*, 2010; *Mayer and Haimberger*, 2012; *Liu et al*., 2015].

### 2.2 Data Sets

The atmospheric total energy transport and tendency are from the ECMWF (European Centre for Medium‐Range Weather Forecasts) Interim reanalysis (ERAINT) [*Dee et al*., 2011; *Berrisford et al*., 2011]. The TOA radiation fluxes prior to the Clouds and the Earth's Radiant Energy System (CERES) era are reconstructed based on *Allan et al*. [2014] and *Liu et al*. [2015] and combined with the CERES EBAF (version 2.8) data from March 2000. The individual satellite Terra and Aqua data (SSF1deg Ed3A) [*Loeb et al*., 2012] (2000–2015) and the Earth Radiation Budget Satellite (ERBS) scanner data (1985–1989) [e.g., *Wielicki et al*., 2002] are also used for further validation. By reconstructing the TOA radiation fluxes using satellite data and the ERAINT reanalysis before the CERES era, and combining with the CERES observations, the TOA radiation fluxes from 1985 to present can be obtained [*Allan et al*., 2014; *Liu et al*., 2015]. The global area mean net TOA radiation flux is anchored to the latest estimation of Earth heat uptake of 0.59 Wm^−2^ over 2006–2013, with 0.49 Wm^−2^ (7.9 × 10^21^ J/yr) by the ocean from 0 to 2000 m [*Roemmich et al*., 2015], 0.07 Wm^−2^ by the deeper ocean [*Purkey and Johnson*, 2010; *Desbruyères et al*., 2016], and 0.03 Wm^−2^ by melting ice, warming land, and an increasingly warmer and moister atmosphere [*Loeb et al*., 2012; *Johnson et al*., 2016; *Stephens et al*., 2016].

The OAFLUX makes use of the optimal blending of satellite retrievals and three atmospheric reanalyses [*Yu et al*., 2008]. The daily fluxes are computed from the optimally estimated variables using the COARE (Coupled Ocean Atmosphere Response Experiment) bulk flux algorithm 3.0 [*Fairall et al*., 2003]. Therefore, OAFLUX is a combined observation‐reanalysis based product. The comparison showed that the differences between OAFLUX and NWP products can be as large as 20–30 Wm^−2^ in the subtropical oceans [*Yu et al*., 2008], and the mean net heat flux into the free ocean is about 25 Wm^−2^ for the combined OAFLUX and ISCCP (International Satellite Cloud Climatology Project) product [see Figure 4 of *Yu et al*., 2007], which is out of the balance. The flux corrections are used by GODAS and ORAS4, where the surface fluxes (momentum, heat and freshwater) from atmospheric reanalyses of NCEP (National Centers for Environmental Prediction) (for GODAS [*Behringer*, 2007]) and ERA‐Interim (for ORAS4 [*Balmaseda et al*., 2013]) are applied directly to the ocean surface, along with a surface relaxation of SST toward an observational product to prevent model drift. The bulk flux forcing is used by C‐GLORS05v3, where turbulent fluxes (heat, water, and momentum) are derived from bulk formula [*Large and Yeager*, 2009] using a prescribed atmospheric state from ERA‐Interim and the model's SST, which may also be affected by data assimilation [*Storto et al*., 2014]. The chosen atmospheric state also includes precipitation and runoff data, and radiative (downward shortwave and longwave) fluxes [*Valdivieso et al*., 2015].

To investigate uncertainty in the surface energy flux methodology, the spread of the multiannual spatial and zonal mean surface fluxes is also investigated using five atmospheric reanalysis data sets: ERAINT, ERA20C (ERA‐CLIM: European Reanalysis of Global Climate Observations [*Poli et al*., 2013]), JRA55 (the Japanese 55 year Reanalysis [*Kobayashi et al*., 2015]), JRA55C [*Kobayashi et al*., 2014], and MERRA (Modern Era‐Retrospective Analysis for Research and Applications [*Rienecker et al*., 2011]). Surface fluxes generated directly by the reanalyses are used in addition to the derived fluxes computed from atmospheric energy tendencies and transports and TOA satellite data. A four‐dimensional variational analysis is used in the ERA‐Interim and JRA55 reanalyses, and a three‐dimensional variational data assimilation in MERRA, where data from the full observing system covering the global atmosphere are assimilated. JRA55C assimilates only conventional surface and upper air observations without the use of satellite observations, using the same data assimilation system as the JRA55. ERA20C assimilates observations of surface pressure and surface marine winds; SST, sea ice, and realistic radiative forcings are prescribed [*Poli et al*., 2013]. All data sets are listed in Table 1 with some brief descriptions.

**Table 1:** Data Sets

Data set	Period (in this study)	Resolution	References
CERES EBAF v2.8	2000–2015		
Terra (SSF1deg‐month Ed3A)	2000–2015	1.0° × 1.0°	*Loeb et al.* [2012]
Aqua (SSF1deg‐month Ed3A)	2002–2015		
ERBS	1985–1989	2.5° × 2.5°	*Wielicki et al.* [2002]
OAFLUX	2000–2015	1.0° × 1.0°	*Yu et al.* [2008]
Buoy data			
TAO/TRITON			*McPhaden et al.* [1998]
RAMA	1989–2015		*McPhaden et al.* [2009]
PIRATA			*Bourles et al.* [2008]
UPSCALE	1985–2011	0.35° × 0.23°	*Mizielinski et al.* [2014]
Reconstruction			
TOA: F_T_	1985–2015	0.7° × 0.7°	*Allan et al.* [2014]
Surface: F_s_			*Liu et al.* [2015]
ERA‐Interim (ERAINT)	1985–2015	0.7° × 0.7°	*Dee et al.* [2011]
ERA‐CLIM (ERA20C)	1985–2010	0.7° × 0.7°	*Poli et al.* [2013]
JRA55	1985–2014	1.25° × 1.25°	*Kobayashi et al.* [2015]
JRA55C	1985–2012	1.25° × 1.25°	*Kobayashi et al.* [2014]
MERRA	1985–2014	0.7° × 0.5°	*Rienecker et al.* [2011]
C‐GLORS05V3	1993–2011	1.0° × 1.0°	*Storto et al.* [2014]
ORAS4	1993–2009	1.0° × 1.0°	*Balmaseda et al.* [2013]
GODAS	1993–2011	1.0° × 1.0°	*Behringer* [2007]

### 2.3 Improved Land Surface Energy Flux Reconstruction

Motivated by unrealistic magnitudes and patterns of estimated surface energy fluxes over land from the right‐hand side of equation (1), *Liu et al*. [2015] proposed a novel method to directly estimate *F*_*s*_ over land, which could in return be used to adjust the field of atmospheric energy divergences (horizontal transport out of the atmospheric column, see equation (1)). This was achieved by generating physically based reconstructions of land surface fluxes based upon detailed modeling and using an energy budget approach. Using a five ensemble member high‐resolution atmospheric model UPSCALE [*Mizielinski et al*., 2014], the simple relation between the global land area mean surface flux *F*_*s*_ and temperature change rate $\frac{\Delta T}{\Delta t}$ ( $F_{s\;}=C\frac{\Delta T}{\Delta t}+\varepsilon ,$ *C* is the effective mean surface land heat capacity, and *ε* is a constant indicating the energy flux penetrating beneath the surface layer) was established by *Liu et al*. [2015]. This was then applied to the ERA‐Interim monthly land surface temperature changes to reconstruct the monthly global mean land surface fluxes. The land surface model in the UPSCALE simulations is JULES (Joint UK Land Environment Simulator), which has an explicit representation of the surface energy balance for vegetation, capturing the weak coupling that exists between the canopy and underlying soil [*Best et al*., 2011].

Since the heat capacity depends upon soil type and moisture content, inaccurate representation of regional relationships between heat flux and temperature change will reduce the realism of the estimated regional changes in surface flux. Therefore, the global method of *Liu et al*. [2015] is modified in this study to include these spatial variations. The five UPSCALE ensemble members are used to derive the seasonal relations, $F_{s}-F_{\text{snow}}=C\frac{\Delta T}{\Delta t}+\varepsilon$, at each grid point with the additional consideration of energy required for snowmelt (*F*_snow_). $\frac{\Delta T}{\Delta t}$ is calculated from consecutive months; for example, the climatology of *F*_*s*_ − *F*_snow_ in April will correlate with $\frac{\Delta T}{\Delta t}$ calculated from the climatology difference between April and March, so the effective land heat capacity *C* and the constant *ε* are calculated by regression using the seasonal climatologies of *F*_*s*_ − *F*_snow_ and $\frac{\Delta T}{\Delta t}$. Five sets of (*C* , *ε*) are derived from the five UPSCALE ensemble members after interpolating the UPSCALE data to the ERA‐Interim grid. They are then applied to the ERA‐Interim simulated $\frac{\Delta T}{\Delta t}$ and *F*_snow_ to estimate *F*_*s*_ over land. The *F*_snow_ from ERA‐Interim has discontinuities between 1992 and 1993 and particularly between 2003 and 2004 (Figure S1 in the supporting information) which are homogenized by adding seasonal anomalies for 1985–1992 and 1993–2003 to the mean seasonal climatologies for 2004–2015. Based on *Beltrami et al*. [2002], the mean net land surface flux is anchored to 0.08 Wm^−2^ over 1985–2012. The purpose of this methodology is to capture the basic spatial relationships between surface fluxes and soil heating depicted by the high‐resolution simulations in order to improve the realism of reconstructed fluxes over land, rather than to explicitly and accurately represent them. To conserve energy, a corresponding adjustment in ocean surface fluxes of opposite sign is required. For example, for the multiannual mean (2001–2005), 3.3 Wm^−2^ is added to land fluxes and 1.2 Wm^−2^ is removed from the global oceans. The land surface flux adjustments are sensitive to surface/soil parameters, such as the thermal conductivity, surface albedo, snow, and ice cover which may not be realistically represented by JULES. As such, substantial uncertainty remains as will be discussed in section 3.4.

### 2.4 Redistribution of the Land Surface Flux Adjustment

The mass‐corrected atmospheric energy divergences and tendencies from ERAINT are used to calculate the net surface fluxes [*Trenberth et al*., 1995; *Mayer and Haimberger*, 2012; *Liu et al*., 2015]. The uncertainty of the TOA radiative fluxes is small compared with the uncertainty in the atmospheric energy transport [*Berrisford et al*., 2011]. Therefore, to remove unrealistically large values in land surface fluxes, adjustments are required to change energy divergences from the atmospheric column and by implication correction to the corresponding horizontal energy transports. The implied changes in horizontal atmospheric energy transport between ocean and land therefore also require a corresponding adjustment to ocean surface energy fluxes: essentially the adjustment in land surface flux requires a corresponding adjustment in ocean surface fluxes of opposite sign.

The adjustments to land surface fluxes are redistributed to the ocean in three ways: (i) evenly across the global oceans, (ii) evenly across the oceans but calculated separately in each hemisphere, and (iii) zonally redistributed to the oceans based on the following steps:

1. The global land is divided into 15° latitudinal bands (a 30° band covers Antarctica) as shown in Figure S2.
2. The monthly land surface fluxes derived from the simple energy budget estimates from the regression of simulated surface energy flux and surface temperature tendency are computed at each grid point.
3. The corresponding atmospheric energy divergences and implied ocean‐land horizontal energy transports are adjusted to ensure the land surface fluxes match the energy budget‐based estimates and the area mean adjustment (difference) is computed over the 15° latitudinal bands.
4. Since the global mean atmospheric energy divergence is zero (no energy is created or lost), to keep the global atmospheric energy conserved, the band‐mean divergence adjustment over land is redistributed to the divergence symmetrically over the oceans across a 45° latitudinal band with peak weighting at the corresponding 15° latitudinal band center and exponentially decay away from the center (Figure S2). This is to avoid significant discontinuities in the ocean meridional transport and implicitly assumes some meridional redistribution of energy fluxes. This is essentially equivalent to redistributing the excess or deficit in land surface fluxes over the oceans.
5. The redistribution of energy is applied only in the same hemisphere to retain cross‐equator transport. This is motivated by the minima in energy transport close to the equator and also to retain consistency with the processing of the pre‐CERES satellite data which is considered separately in each hemisphere.

The method to redistribute the excess surface fluxes over land to oceans is imperfect. The transport is dominated by the strong westerlies in the Northern Hemisphere in winter, which supports to some extent our quasi‐zonal redistribution [*Trenberth and Fasullo*, 2013]. However, it is more complicated in summer when land surface temperature is higher than the ocean temperature; regional land‐sea circulation disturbs the quasi‐zonal redistribution assumption, particularly over the monsoon areas.

The phase change of ice over both land and oceans can also affect the net surface fluxes but, aside from snowmelt over land, it is not dealt within this study, due to uncertainties in model simulations and reanalyses and the lack of observations.

## 3 Results

### 3.1 Update of the TOA Radiation Flux

**Figure 1:**
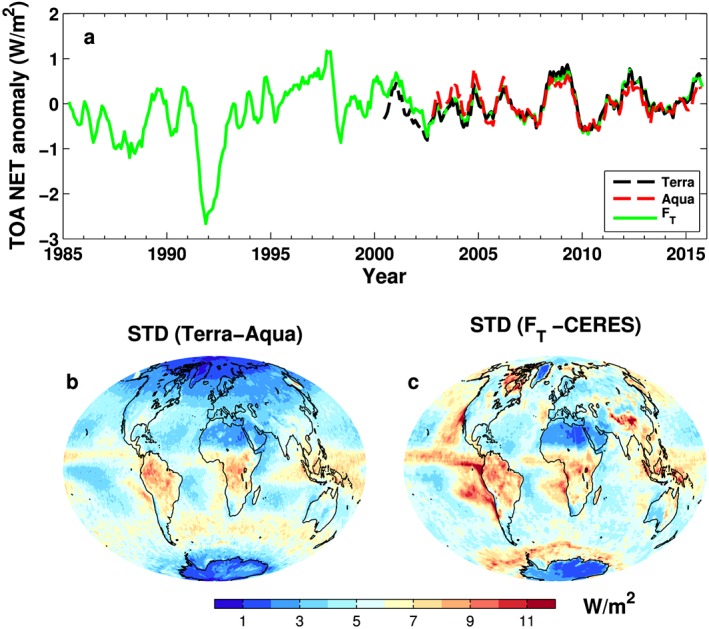
(a) The top of atmosphere net radiation anomaly time series, with reference period 2003–2014. All lines are 6 month running means. Spatial distribution of standard deviations from monthly anomaly differences over 2003–2014: (b) between Terra and Aqua and (c) between reconstruction *F*_*T*_ and CERES.

Figure 1a displays deseasonalized global mean net TOA radiation flux anomalies (relative to 2003–2014) from the reconstruction [*Allan et al*., 2014] and Terra and Aqua CERES data (SSF1deg ED3A). The correlation coefficient between Terra and Aqua time series is 0.92, and the standard deviation of the anomaly differences is 0.26 Wm^−2^ from monthly means over 2003–2014 and 0.1 Wm^−2^ from annual means. *Johnson et al*. [2016] estimated an uncertainty of ±0.1 Wm^−2^ at 95% confidence level based on consideration of ocean heating trend uncertainty, but this applies to the 2005–2015 period. We choose to anchor the CERES record to the 2006–2013 period, based on the analysis of *Roemmich et al*. [2015]. Our reason for choosing this period is that the ocean heat content data appear to show linear increases [*Stephens et al*., 2016] with larger increases thereafter that are not present in the CERES data [*Johnson et al*., 2016]. Our estimate produces a slightly lower (~0.59 Wm^−2^) net flux compared with 0.71 Wm^−2^ of *Johnson et al*. [2016].

The spatial distributions of the monthly anomaly difference standard deviations are calculated between Terra and Aqua (Figure 1b) over 2003–2014. The differences are mainly from the contrasting sampling, since they are imaging approximately 3 h apart the same area at the equator, and the large differences over tropical convective regions are linked to the strong diurnal cycles of clouds. The Terra and Aqua orbits cross one another at 70°N and are roughly 6 h apart at 60°S, which is also reflected in Figure 1b with smaller standard deviations in the Northern Hemisphere compared to the Southern Hemisphere.

To estimate the regional flux uncertainty prior to the CERES period, we applied the methodology of *Allan et al*. [2014] using ERA‐Interim monthly anomalies combined with the CERES climatological seasonal cycle but instead applied to the 2003–2014 CERES period. Differences between the reconstructed *F*_*T*_ and CERES data therefore show the uncertainty (Figure 1c) in applying regional anomalies from ERAINT in the reconstruction of fluxes and may be used as a proxy for regional uncertainty prior to the CERES period. The standard deviation of differences between Terra and Aqua (global average of 4.7 Wm^−2^ and grid point maxima of ~11 Wm^−2^) is smaller than that between *F*_*T*_ and CERES (global average of 5.7 Wm^−2^ and grid point maxima of ~20 Wm^−2^), particularly over the eastern Pacific area and the Southern Ocean. The corresponding global mean standard deviations using annual means are 1.5 Wm^−2^ (up to a maximum of 6 Wm^−2^) and 2.1 Wm^−2^ (up to a maximum of 10 Wm^−2^), indicating that the regional uncertainty in TOA flux reconstruction prior to the CERES period is larger than the discrepancy between Terra and Aqua measurements due to sampling time differences.

### 3.2 Evaluation of Land Surface Flux Reconstruction

Although the updated land surface flux reconstruction method is not based directly on observations, it takes advantage of the physical relationships represented by the JULES model which includes the interaction between the canopy and the underlying soil [*Best et al*., 2011]. Therefore, the regressed grid point effective land heat capacity represents more realistically the land surface features and seasonal variability than the global mean values when applied to the ERA‐Interim snowmelt and temperature change.

**Figure 2:**
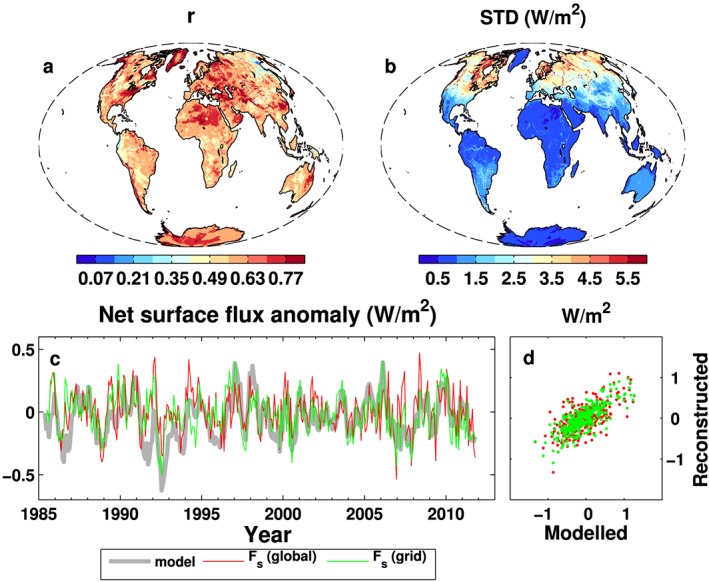
(a) The anomaly correlation coefficient and (b) the standard deviation spatial distributions between the modeled and reconstructed surface flux anomalies using data from one UPSCALE member. (c) Anomaly time series of *F*_*s*_ from the model and reconstructions using the global mean relation and the relation at each grid point with the consideration of the snowmelt. (d) The scatterplot between the modeled and reconstructed anomalies. The reference period is 2001–2005. Lines in Figure 2c are 6 month running means.

To assess the updated method for estimating land surface energy flux described in section 2, the reconstructed land surface energy flux anomalies are compared with the raw model simulations of surface energy flux from one of the five UPSCALE members. The data period is from 1985–2011 and deseasonalized anomalies are computed with respect to the 2001–2005 reference period. The correlation coefficients (*r*) between anomalies at each grid point are plotted in Figure 2a: *r* > 0.5 over 90% of land grid points with lower correlation coefficients over a small region of northeast Asia which may also be affected by a number of factors including snowmelt in spring. The standard deviations (STDs) between the modeled and reconstructed net surface fluxes (Figure 2b) are large over middle and high northern latitudes, and this may be related to the melting and freezing of ice. The global mean STD is 2.0 Wm^−2^, slightly smaller than 2.3 Wm^−2^ from the previous method of *Liu et al*. [2015].

The global land mean anomaly time series (6 month running means) from the model and the reconstruction (*F*_*s*_ ) are plotted in Figure 2c, and the corresponding anomaly scatterplot is in Figure 2d. The red line is reproduced from *Liu et al*. [2015] using relations between the global land area mean net surface fluxes and the surface temperature change rates, and the green line is the updated reconstruction using grid point relations with the consideration of snowmelt. The anomaly correlation coefficients increase from 0.65 to 0.78 (Table S1), demonstrating the benefit of the modified energy budget relationship at grid points and accounting for snowmelt. After the reconstructed variability (red and green) are multiplied by the ratio of the standard deviation between modeled and reconstructed monthly flux anomaly running means (values in Table S1), the reconstructions are able to adequately capture the directly modeled variations. Negative anomalies in land surface flux following the Mount Pinatubo eruption (Figure 2c) are marginally better represented when account is taken of grid point relationships and snowmelt. The corresponding correlation coefficients (*r* = 0.74–0.78), standard deviations ratio (1.8–2.1), and the global land area mean effective land heat capacity (28.8 ± 2.5 to 29.3 ± 2.9 Wm^−2^ K^−1^ day) from the five UPSCALE members are listed in Table S1. The standard deviation ratios from 6 month anomaly running means are used to inflate the reconstructed anomalies since the reconstruction process using the seasonal effective land heat capacity artificially smoothes the reconstructed values.

### 3.3 Evaluation of Adjusted Ocean Surface Fluxes and Heat Transports

Since surface fluxes directly simulated by the reanalysis models are not used in our estimations, errors in cloud fields and other factors will not directly influence surface fluxes for example through shortwave radiation biases [*Slater*, 2016]. However, cloud errors may indeed influence the accuracy of other reanalysis fields such as the subcloud temperature which can potentially influence the quality of horizontal energy transport calculations. Regionally, biases in temperature, moisture, and wind fields can generate inaccurate energy transports, and this is likely to be larger for regions of complex coastline or topography. In order to check the quality of the estimated net surface fluxes, the turbulent energy (latent heat plus sensible heat) fluxes from OAFLUX are used here for comparison. Our turbulent energy fluxes are derived by computing the difference between the estimated net surface fluxes and the radiative surface fluxes from CERES EBAF satellite estimates. The surface radiative flux estimates are constrained by satellite radiation budget and cloud property data, and although they will contain biases related to the additional input data required for the radiative transfer calculations, they have been carefully validated [*Rutan et al*., 2001; *Kato et al*., 2013] and are considered to be more reliable than surface turbulent flux observations.

**Figure 3:**
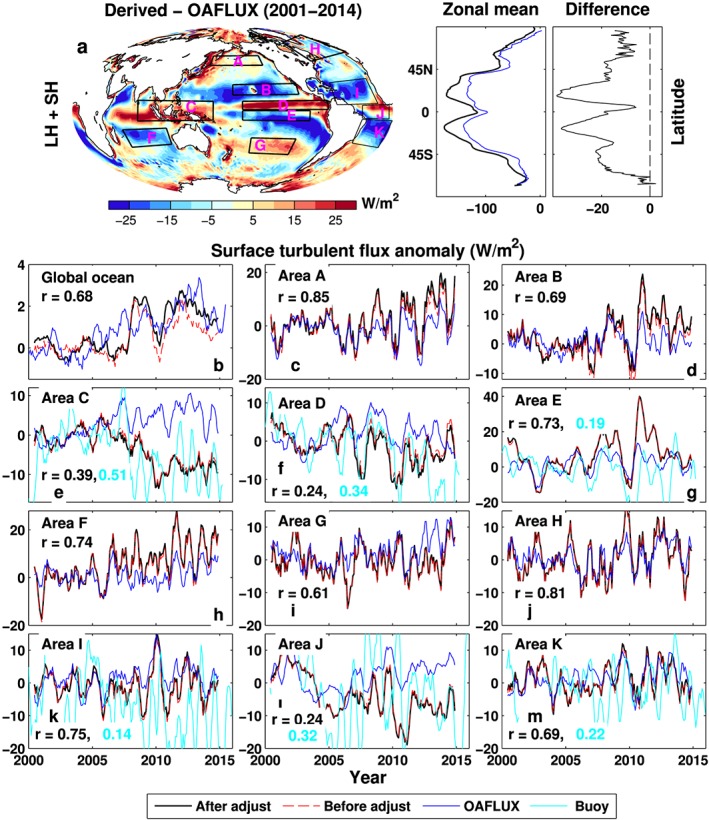
Comparison of turbulent energy flux (positive downward) anomalies between the derived (estimated net surface flux minus surface radiative flux from CERES EBAF) and those from OAFLUX. (a) Multiannual mean (2001–2014) turbulent energy flux difference map, zonal mean, and zonal mean difference. The global ocean mean bias of 20 Wm^−2^ is removed from the global map; (b–m) deseasonalized turbulent energy flux anomaly variability over different regions defined in Figure 3a and Table S2. The reference period is 2001–2005. Turbulent energy fluxes derived from the net surface fluxes before the land surface flux adjustment are also plotted (red dashed line). The buoy data are composite observations. The correlation coefficients and the number of buoy stations in each region are listed in Table S2. All lines are 6 month running means.

The multiannual mean (2001–2014) maps of turbulent energy flux (positive downward) differences between our calculations and the Woods Hole Oceanographic Institution (WHOI) data are shown in Figure 3a, together with their zonal mean distributions and the differences. This shows that the turbulent energy fluxes of WHOI are systematically lower than our estimations, which may explain why the mean net surface flux is so high (~25 Wm^−2^) in combined OAFLUX and ISCCP product [*Yu et al*., 2007]. In order to ensure consistent comparison while maintaining the spatial distribution properties of both data sets, a mean bias of 20 Wm^−2^ is removed from the difference map (two‐dimensional plot of Figure 3a). This shows that the turbulent fluxes from our estimation are larger in high latitudes and the tropics but smaller over the subtropics than those from OAFLUX; maximum differences are up to 25 Wm^−2^.

Variability in turbulent fluxes are now compared over regions A–K defined in Figure 3a and Table S2. Deseasonalized anomalies (relative to the 2001–2005 period) are computed over the global ocean and the 11 ocean regions (Figures 3b–3m). The derived turbulent energy fluxes before and after the land surface flux constraint are plotted: both show good agreement over regions A–K, but there are some differences over the global ocean (Figure 3b). The correlation coefficients (*r*_1_ in Table S2) between turbulent flux anomalies from the OAFLUX and the derived ones after the land surface flux constraint are displayed. Other correlations between different data sets considered are also listed in Table S2. The anomaly correlation coefficient *r*_1_ over the global ocean is improved from 0.55 (Table S2) to 0.68 before and after the land surface flux constraint, but they are almost the same over regions A–K, implying the land surface constraint has little impact over these regions on the level of correlations with OAFLUX. The comparison between the derived turbulent energy flux variability and those from OAFLUX is generally good for selected regions, with time series correlation coefficients *r*_1_ ≥ 0.61, except for regions C, D, and J (Figures 3e, 3f, and 3l). Region C covers part of the Indian Ocean, the Indonesian throughflow, and the tropical warm pool (Figure 3e), where the anomaly time series diverge after 2006 and the correlation coefficient ***r***_1_ = 0.39. However, the composite turbulent energy flux variability from 25 TAO/TRITON buoy stations in region C supports our estimation, and the correlation coefficient between them is ***r***_3_ = 0.51, while there is a lack of correlation between the OAFLUX and buoy data (***r***_5_ = 0.04, see Table S2). In general, the correlations between our estimation and buoy data are marginally better than those between OAFLUX and buoy data over all selected regions where buoy data are available (Figure 3 and Table S2), confirming the improvement of the turbulent flux estimation from our product over the OAFLUX on this aspect.

**Figure 4:**
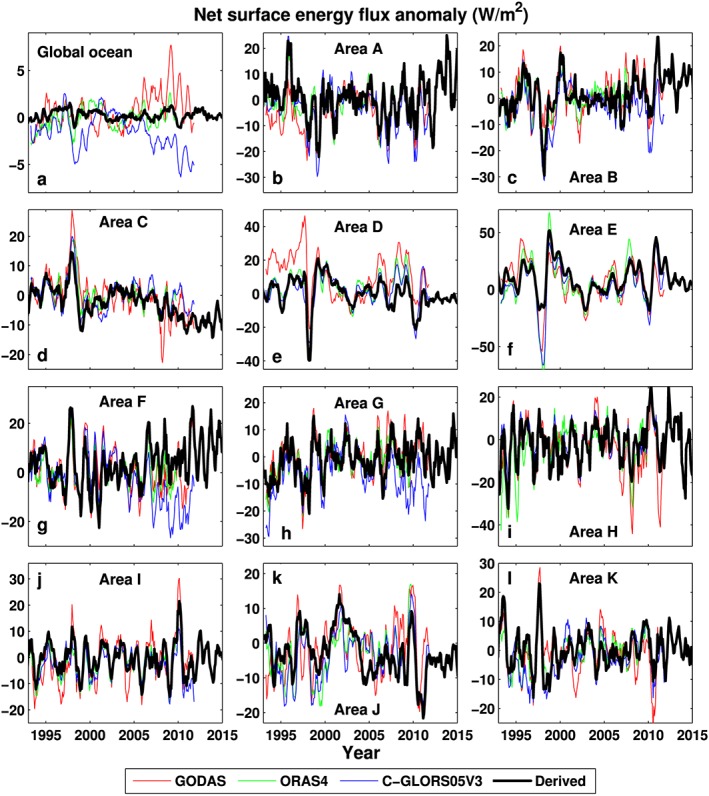
Comparison of derived net surface energy flux anomalies and GODAS, ORAS4, and C‐GLORS05V3 reanalyses for (a) global ocean and (b–l) 11 selected regions shown in Figure 3a. The reference period is 2001–2005. The correlation coefficients are listed in Table S3. All lines are 6 month running means.

The estimated net surface flux variabilities are also compared with those from the ocean reanalyses, including GODAS, ORAS4, and C‐GLORS05V3 (Figure 4). Although the correlation coefficients are low between our global ocean estimation and GODAS and C‐GLORS05V3, the correlation with ORAS4 shows consistent anomaly variations (Figure 4a), which may be partly because the ERA‐Interim surface fluxes are used in ORAS4. There is good agreements across these four data sets over 11 selected regions (Figures 4b–4l) with 92% of ***r*** ≥ 0.6 (Table S3), but do bear in mind that both data sets of ORAS4 and C‐GLORS05V3 are not independent from ERA‐Interim.

As a further indirect evaluation of the surface fluxes we estimate meridional ocean heat transports using methods similar to *Trenberth and Fasullo* [2017], testing our product and the alternative methods described in section 2.4 which are also generated as intermediate products. The meridional ocean heat transports are inferred from the net surface heat fluxes for several cases: (i) without land surface adjustment (*No adjustment*), (ii) redistribute the adjustment evenly to the global ocean (*Whole ocean*), (iii) even distribution to the oceans in the same hemisphere (Southern Hemisphere and Northern Hemisphere oceans, hereafter *SH and NH oceans*), and (iv) redistribute the adjustment zonally using a weighting function (see Figure S2). Details of the multiannual mean (2006–2013) adjustment of the land surface flux and redistribution over the ocean are illustrated in Figure S2.

**Figure 5:**
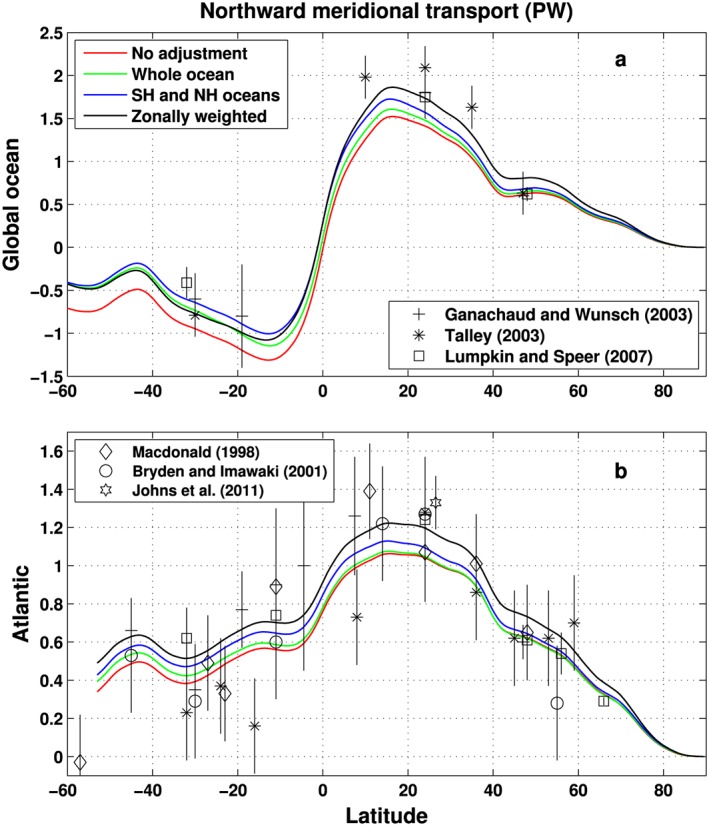
Multiannual mean (2006–2013) northward total meridional ocean heat transports (unit is PW) for (a) the global ocean and (b) the Atlantic derived from the reconstruction (lines) and estimated from observations (symbols, error bars show one standard deviation). Reconstructions are inferred from ocean heating estimated from *Roemmich et al*. [2015] combined with the reconstructed ocean surface fluxes applying the following methods: without land surface flux adjustment (*No adjustment*, red line); the adjustment is applied evenly to the global ocean (*Whole ocean*, green line); the adjustment is applied evenly to the oceans in the same hemisphere (*SH and NH oceans*, blue line); and the adjustment is redistributed zonally using the weighting function (*Zonally weighted*, black line).

The inferred meridional ocean heat transports [*Valdivieso et al*., 2015], together with the observed oceanic heat transports, are plotted in Figure 5. In our calculations, the ocean heat transports are calculated by integrating the net surface heat fluxes over oceans from the north and considering the zonal mean ocean heating estimated from *Roemmich et al*. [2015] (Figure S3a). Since the CERES TOA net radiation flux is anchored to the total ocean heating of *Roemmich et al*. [2015] and the meridional ocean heat transport is calculated over the same period (2006–2013), considering the atmosphere has a comparatively small heat capacity, the total surface energy into the ocean is approximately balanced by the total ocean heating over this period. Therefore, the integration residual at the South Pole is close to zero, and the effect of the integration direction (from north to south or from the south to north) on the meridional ocean heat transports can be neglected.

The ocean heating only has a small effect on the meridional ocean heat transport calculation in the Northern Hemisphere due to the weak oceanic heating there but has some effect in the Southern Hemisphere, particularly in the southern ocean, due to the significant observed oceanic heating [*Roemmich et al*., 2015] (Figure S3b). The data from *Ganachaud and Wunsch* [2003] and *Lumpkin and Speer* [2007] are from the WOCE‐based inverse model results. The estimates from *Macdonald* [1998] are also from the box inversions using pre‐WOCE zonal hydrographic sections. *Talley* [2003] estimates are based on the absolute geostrophic velocity analyses for coast‐to‐coast hydrographic sections and accompanying temperature [*Reid*, 1994, 1997]. The data from *Bryden and Imawaki* [2001] are based on the hydrographic section measurements, and the RAPID estimate is from *Johns et al*. [2011] for the period from April 2004 to October 2007. The error bars are one standard deviation.

For the global ocean, the transport from the current method (labeled as “*Zonally weighted*”, black line, where the ocean flux is zonally adjusted) is systematically higher and closer to the observations than the other methods in the Northern Hemisphere. The southward transport from the net surface fluxes inferred from the mass‐corrected total atmospheric energy divergences, but without any land surface flux adjustment (labeled as “*No adjustment*”, red line), is higher than other methods in the Southern Hemisphere. For the Atlantic, the observations are quite spread, but there are more observations close to the transport estimated from the current method (black line) between the equator and 40°N. To quantify this, the differences between the inferred meridional ocean heat transports and the observations are calculated at the same latitude and the mean bias and standard deviations of the monthly differences for each data sets are computed as shown in Table S4.

The transports from all redistribution methods are superior to the transport estimated from *“No adjustment”* (last column of Table S4). For the global ocean, a mean bias of −0.37 PW (petawatts) and standard deviation of the differences is 0.26 PW when no surface flux adjustment is applied, and the corresponding values are −0.12 PW bias and 0.22 PW standard deviation for differences based on the zonal redistribution method. Compared with observations, the zonal redistribution method is also marginally superior to the other two redistribution methods for the global ocean but when considering the Atlantic there is little difference between all four methods (Table S4). The analysis across all data sets presented in Table S4 indicates that the zonal redistribution method is marginally better than the hemispheric or global redistribution methods, and this is also true for the majority of the individual data sets.

The estimated multiannual mean (2004–2013) Atlantic heat transport at 26.5°N is about 1.16 PW, which is closer to the RAPID observation [*Frajka‐Williams*, 2015] of 1.23 PW than the estimation of 1.0 PW by *Trenberth and Fasullo* [2017] over the same period, confirming the improvement of the inferred transport after the land surface flux adjustment.

### 3.4 Uncertainty Investigation

The spread of surface energy flux from different observationally based data sets is substantial [*Macdonald and Baringer*, 2013; *Josey et al*., 2013; *Valdivieso et al*., 2015] and relates to a number of deficiencies in observing systems and models being applied. Uncertainties in the reconstruction method utilized in the present study are determined by the accuracy and stability of the TOA radiation flux and by the reanalyzed atmospheric energy divergences (horizontal transports) and tendencies.

#### 3.4.1 Top of Atmosphere Radiation

*Loeb et al*. [2012] estimated an uncertainty of ±0.43 Wm^−2^ at the 90% confidence level in global mean TOA radiation, primarily determined by ocean heat uptake uncertainty of ±0.38 Wm^−2^ over the period 2005–2010. Capitalizing on an improved and extended ocean heating data set, *Johnson et al*. [2016] estimate TOA energy flux uncertainty of ±0.1 Wm^−2^ at the 95% confidence level. Based upon these uncertainty differences and the potential calibration drift uncertainties of order 0.2 Wm^−2^/decade, we estimate the annual 90% confidence uncertainty range for the period 2000–2015 as ±0.14 Wm^−2^ by adding in quadrature the 0.1 Wm^−2^ relating to 2005–2015 ocean heating and 0.1 Wm^−2^ relating to CERES stability uncertainty. Prior to the CERES period, an additional uncertainty of ±0.24 Wm^−2^ [*Allan et al*., 2014] relating to homogeneity adjustment increases total uncertainty to ±0.38 Wm^−2^. Regional uncertainty in TOA net radiation is estimated from the satellite data and the reconstruction method (please refer to section 3.1 and Figure 1).

#### 3.4.2 Estimating Regional Uncertainty in Surface Flux

**Figure 6:**
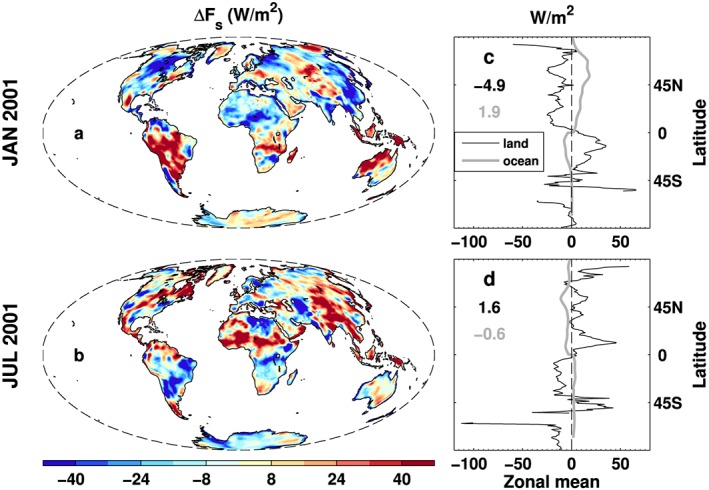
The surface flux differences (flux without land constraint minus flux with land constraint) (a) in January 2001 and (b) in July 2001. (c and d) The corresponding zonal means over land and oceans. Global land and global ocean area means are displayed in Figures 6c and 6d as well.

The land *F*_*s*_ difference before and after the energy budget constraint can provide some clues on the characteristics and possible magnitudes of the regional uncertainties. To illustrate the magnitude of the monthly land surface flux constraint, *F*_*s*_ differences before and after the constraint in January and July 2001 are plotted in Figure 6, together with the zonal mean differences in the right column and the corresponding zonal mean differences when adjusted energy fluxes are distributed over the oceans (gray line). The global land and the global ocean area mean adjustments are displayed in the zonal mean plot. The land constraint exhibits different seasonal effects (Figures 6a and 6b) which are also shown in the January and July climatology differences (not shown here). The local differences are substantial over land (up to around 40 Wm^−2^ in magnitude), which is mainly from the atmospheric energy divergence (transport) and is not sensitive to the land surface flux reconstruction method. This is not only due to the bias in the atmospheric energy divergence from ERA‐Interim reanalysis but also due to the deficiency of the mass correction algorithm currently employed; a more comprehensive algorithm including the moisture transport correction should be investigated in future studies. It is plausible that a similar magnitude uncertainty applies regionally over the ocean. However, complex coastline and terrain are likely to increase errors in horizontal transport over land so that the differences depicted in Figure 6 are considered an upper estimate of expected monthly regional surface energy flux uncertainty over the ocean. Also, the flux adjustment is expected to substantially reduce bias based upon the physically constrained energy budget approach.

In January, over most land areas, the flux excess is negative and the zonal mean in the Northern Hemisphere is between 0 and −25 Wm^−2^ except for larger values near the North Pole. The zonal mean differences are positive over the latitude band 0°–45°S and reach 25 Wm^−2^ to the south of the equator. The overall global land area mean flux discrepancy is −4.9 Wm^−2^ (negative values denote energy flux must be added to the land surface and removed from the adjacent oceans). This difference is redistributed over the oceans to ensure global energy balance (see details below). The deficit over land is consistent with *Trenberth and Fasullo* [2013]. It can be explained by the systematic underestimation of the atmospheric energy transport from ocean to land in ERA‐Interim data, primarily explained by the latent heat term since sensible heat biases compensate between regions. In the Northern Hemisphere winter, the total energy transport is systematically lower than the radiative energy loss from surface to space of the CERES EBAF measurement. So the global mean land surface flux bias may be from the systematic underestimation of the water vapor transport from ocean to land [*Demory et al*., 2013]; however, the large regional errors in sensible heat related to the biases in the temperature field cannot be ruled out [*Trenberth and Stepaniak*, 2003]. The zonal mean difference over the oceans is quite smooth while the mean redistributed excess is 1.9 Wm^−2^, smaller in magnitude than over land due to the larger ocean area. The peak around 55°N is because of the relatively larger land area. In July, the spatial pattern and the zonal mean of the excess fluxes show opposite distributions compared with January. The overall zonal mean differences are generally less than 20 Wm^−2^ in magnitude except for the peaks at high latitudes and near the Equator. The global land area mean flux excess (1.6 Wm^−2^) is removed and added to the global ocean (−0.6 Wm^−2^ denotes a surface flux deficit over ocean before adjustment). The warmer land in summer generates land‐sea circulations, particularly over monsoon areas, transporting dry static energy (DSE) from land to ocean and moisture from ocean to land as latent energy (LE). The cancellation between very large LE and DSE transports leads to a small net transport from land to ocean [*Trenberth and Stepaniak*, 2003], so any errors in the LE and DSE will cause biases [*Trenberth and Fasullo*, 2013].

**Figure 7:**
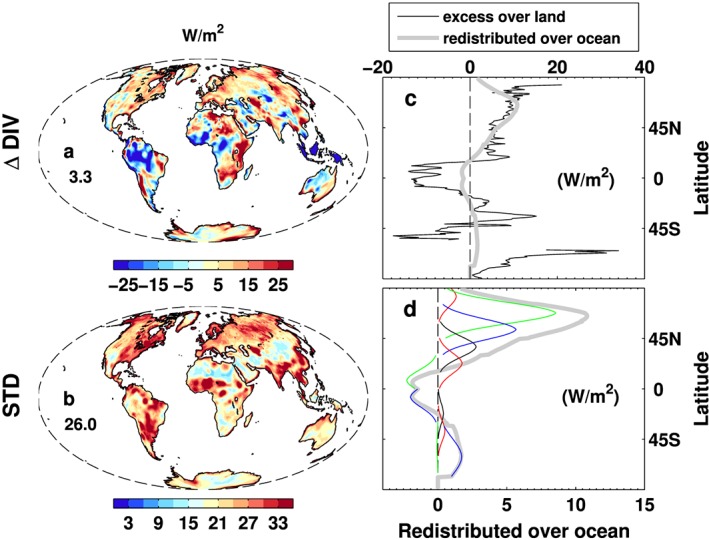
(a) Multiannual (2001–2005) mean monthly divergence difference before and after the constraint, (b) standard deviation of the monthly divergence differences, (c) zonal mean excess divergence over land and the redistribution over the oceans, and (d) redistributions over oceans from 11 latitudinal land bands and the colored curves represent the corresponding redistribution over oceans (see Figures S2). The global land means are also displayed in Figures 7a and 7b. Units are all in W/m^2^.

The multiannual mean (2001–2005) atmospheric energy divergence (through horizontal transports) difference before and after the land constraint (Figure 7a) is 3.3 Wm^−2^, meaning the correction reduces energy convergence over land. The standard deviation of monthly differences before and after the land adjustment (Figure 7b) is 26 Wm^−2^ for the global land mean which provides a conservative estimation of the monthly mean regional surface flux uncertainty. The zonal mean distribution and corresponding redistribution over the ocean are shown in Figure 7c. Each color curve represents the redistribution of the zonal mean excess atmospheric energy divergence over land to the divergence over ocean. The curves corresponding to the 11 latitudinal bands can be identified by their peak positions (also see Figure S2). The excess energy over land and the redistribution over the ocean are similar north of 55°N due to comparable land and ocean areas. South of 55°N, the energy redistribution over the ocean is much smaller than over land due to the relatively large ocean area. There are larger divergences before the land flux constraint north of 55°N and for 20–45°S, and large convergence over the tropical land area and between 45°S and 60°S.

Figure 7d is the excess land divergence (Figure 7a) redistribution over oceans from each of the 11 latitudinal bands. The total excess in a 15° latitude band is redistributed symmetrically over a 45° latitude ocean band with peak weighting at the center of the corresponding 15° latitude band center and exponential decay (quasi‐normal) away from the center to avoid any discontinuity at the latitude band boundary. This accounts for redistribution of energy by the atmosphere in a rudimentary way; a more physically based strategy, such as tracking where the energy excess/deficit originates, is beyond the scope of the present study and is not considered essential given the uncertainties in input data and methodology. The overall redistribution (gray line) shows a peak of ~11 Wm^−2^ around 55°N, 2 Wm^−2^ over the tropical oceans, and about 3 Wm^−2^ over the Southern Ocean (40–60°S).

#### 3.4.3 Estimating Mean Uncertainty From Multiple Atmospheric Reanalyses

**Figure 8:**
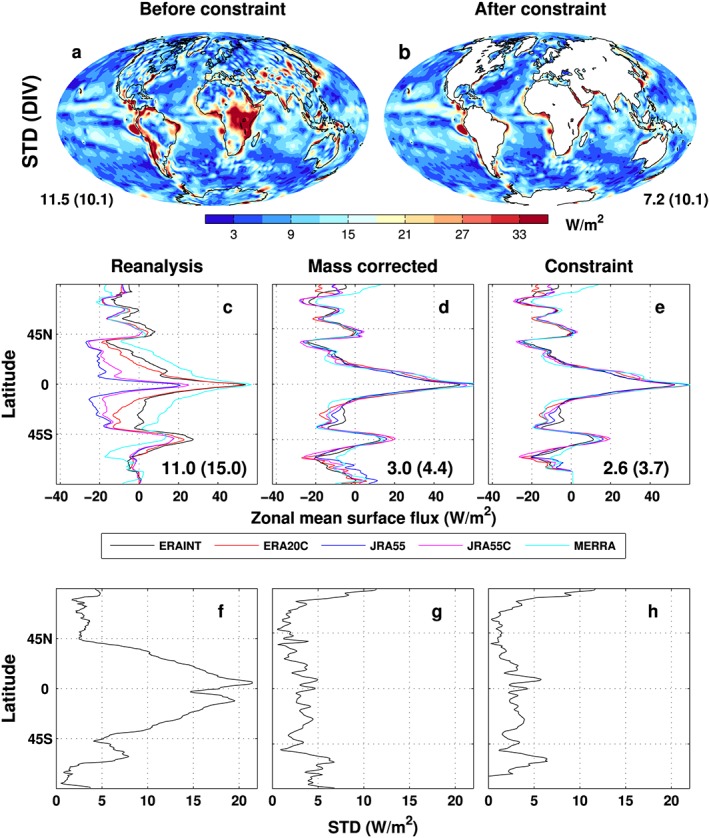
(a and b) Standard deviations of the multiannual mean (2001–2008) mass‐corrected atmospheric energy divergences of five reanalyses (ERAINT, ERA20C, JRA55, JRA55C, and MERRA) before and after land constraint. Values represent global mean standard deviation (global ocean mean in parentheses). Zonal mean global net surface fluxes from atmospheric reanalysis: (c) from model output, (d) from mass‐corrected atmospheric energy divergences without land flux constraints, (e) from mass‐corrected atmospheric energy divergences and land surface flux constraints, and (f–h) from corresponding standard deviations of Figures 8c–8e. The mean of the zonal standard deviations across data sets is displayed in Figures 8c–8e (for ocean only in parentheses).

A primary cause of surface energy flux inaccuracy is likely to originate from the reanalysis‐based atmospheric energy divergences (both model generated and mass‐corrected divergences relating to calculated horizontal energy transport), the uncertainty of which is challenging to quantify. The accuracy of horizontal energy transport depends upon the spatial structure of temperature and moisture and the winds used in the calculation provided by reanalyses. The surface flux uncertainty relating to these factors can be investigated by considering a range of input reanalysis data used in these calculations. Horizontal energy transports are computed from the reanalyses using a consistent methodology to the present study and ensuring atmospheric energy divergences are mass corrected [*Mayer and Haimberger*, 2012; *Liu et al*., 2015]. The STDs of the multiannual mean (2001–2008) mass‐corrected atmospheric energy divergences, with and without a land flux constraint, from five reanalyses (ERAINT, ERA20C, JRA55, JRA55C, and MERRA) are plotted in Figures 8a and 8b. Since ERA20C and JRA55C do not include the assimilation of satellite data, this may increase the range of atmospheric energy divergence estimates since spatial structure in the fields that determine horizontal energy transport are less well constrained than the remaining reanalysis products. It can be seen that before applying the land surface flux constraint (Figure 8a), the regional STD values are large; area mean values are 10.1 Wm^−2^ over the global ocean and 14.8 Wm^−2^ over the global land. The largest STD values are mainly along the west coast of southern America, Africa, and the Kuroshio current area. After the land surface flux constraint is consistently applied (Figure 8b), the STD values are greatly reduced and the global mean value is 7.2 Wm^−2^. However, this reduction is contributed primarily from the land surface fluxes: since the same reconstructed land surface flux estimate is used, the STD in Figure 8b is zero over land. The global ocean mean STD stays roughly the same as before (~10.1 Wm^−2^), suggesting little improvement regionally.

It is also informative to contrast these calculations with the net surface flux estimates provided directly by the reanalysis simulations. Figures 8c–8h show the multiannual (2001–2008) zonal mean surface net fluxes and corresponding average zonal mean STD from five reanalyses. Figure 8c displays global zonal means from the direct model output of ERAINT, ERA20C, JRA55, JRA55C, and MERRA (denoted “Reanalysis”), and the corresponding zonal mean STD is in Figure 8f. The mean STD is 11.0 Wm^−2^, and the large discrepancies are over the tropical region and around 60°S, although they all show similar latitudinal variations. There is a peak STD of 22 Wm^−2^ near the equator. The Reanalysis displayed in Figure 8c is the net surface flux from direct reanalysis model output. It is generated from parametrizations of turbulent and radiative energy fluxes; the radiative transfer model is applied to some parametrized variables, such as cloud, which is partly determined by the model's cloud scheme and may present substantial biases [*Slater*, 2016]. Therefore, agreement between zonal mean distributions in Figure 8c is poor, further justifying the application of our methodology which instead estimates surface energy fluxes as a residual of energy transports, tendencies, and satellite TOA radiation fluxes (Figure 8e).

Surface fluxes are subsequently recalculated using consistent TOA fluxes (section 3.1) and the mass‐corrected atmospheric energy transports. The calculated global zonal mean surface flux (Figure 8d) shows substantially better agreement than from the direct reanalysis model output (Figure 8c). The average zonal mean STD is greatly reduced from 11.0 Wm^−2^ to 3.0 Wm^−2^ and reduces from 15.0 Wm^−2^ to 4.4 Wm^−2^ for the ocean. The zonal mean STD is generally ~1–5 Wm^−2^ but larger near the North Pole (Figure 8g) which may be affected by the sea ice latent heat and merits further study. The land surface flux constraint described in section 2 only has a small effect on the zonal mean flux distribution, and the mean STD is slightly reduced to 2.6 Wm^−2^ (Figure 8e). Unlike for the global analysis (Figures 8a and 8b), when zonal mean of the STD is considered, the land constraint also reduces ocean zonal mean differences (STD = 3.7 Wm^−2^; Figure 8h). This provides a useful although incomplete estimate of the zonal mean uncertainty of the surface flux product described in the present study and indicates consistency in this approach across a range of reanalysis products. Further improvement in the method would require detailed consideration of the errors in the state variables that determine horizontal energy fluxes (e.g., temperature, water vapor, wind speed and direction) at each time step which is beyond the scope of the present work.

### 3.5 Multiannual Mean Energy Trend and Imbalance

**Figure 9:**
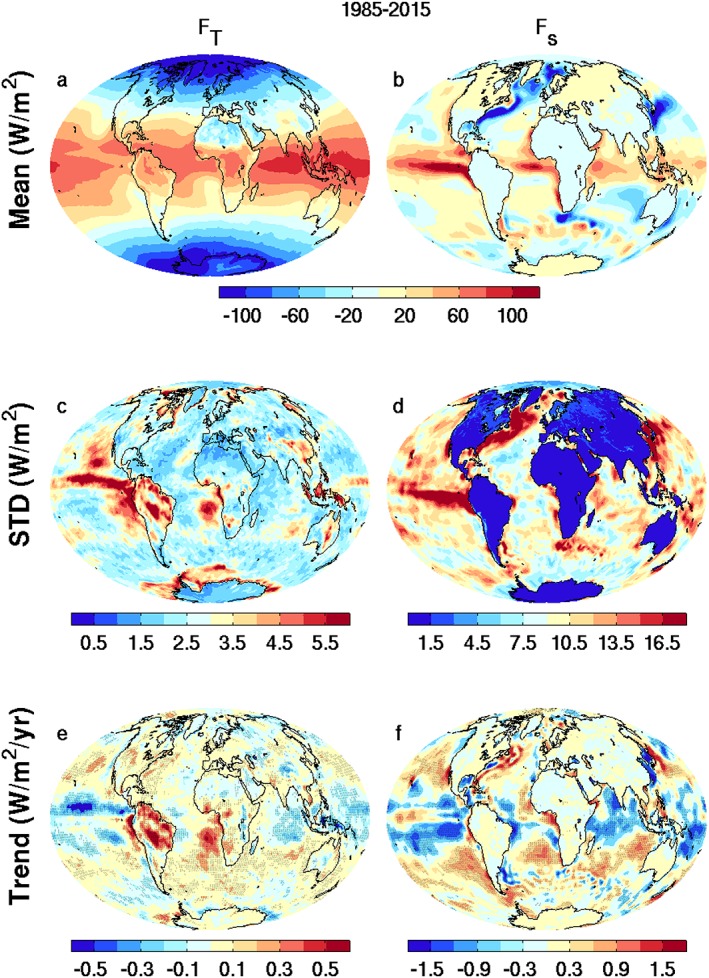
Multiannual means (1985–2015) of (a) net TOA radiation flux and (b) net surface energy flux; standard deviation of annual mean variations at (c) TOA and (d) surface over 1985–2015; the annual mean trends of (e) net TOA radiation flux and (f) net surface energy flux. The stippling shows where the trend is significant using Mann‐Kendall test at significance level of 0.05.

In addition to absolute uncertainty in regional and zonal energy flux, it is also important to gauge the robustness of long‐term global, hemispheric and regional changes in the energy budget determined by internal and externally forced climate variability and change and also by homogeneity issues. For context and to provide a reference period to interpret changes, the multiannual mean (1985–2015) net TOA radiation flux is shown in Figure 9a. The net heating patterns over the tropical area are primarily from the interaction between radiation fluxes and low‐altitude stratocumulus cloud distributions. The lower net downward TOA fluxes over the oceans to the west of southern Africa, southern America, and Australia are due to low‐altitude stratocumulus clouds which contribute a cooling effect on the Earth system. The derived mean net downward surface flux (Figure 9b) is strongly positive into the eastern Pacific tropical oceans due to the interaction between the tropical instability waves [*Willett et al*., 2006] and the equatorial Pacific cold tongue [*Martínez‐Garcia et al*., 2010] where evaporation is suppressed and there is strong shortwave radiation flux reaching the surface. The negative downward fluxes over the Gulf Stream in the North Atlantic and Kuroshio currents in the North Pacific are due to heat and moisture transport from the warm ocean surface to the cold atmosphere above [*Kwon et al*., 2010].

The corresponding standard deviation of the annual mean net TOA radiation fluxes (Figure 9c) is large (>5 Wm^−2^) over the central tropical eastern Pacific due to El Niño Southern Oscillation (ENSO) related variability. Large values (~5 Wm^−2^) near the west coast of southern Africa (Figure 9c) are not detected in the annual mean STD after 2000 (not shown). Regional decadal variation may relate to the reconstruction of the TOA fluxes prior to the CERES era using ERAINT anomaly spatial distributions and the CERES seasonal climatologies. However, changes consistent with the reconstruction are present in differences between CERES (2001–2008) and independent ERBS (1985–1989) satellite data (Figure S4), suggesting this represents real decadal variability due to cloud cover changes [*Norris et al*., 2016; *Zhou et al*., 2016].

As expected, the STD of *F*_*s*_ (Figure 9d) is very small over land since the land heat capacity, which is constrained using the JULES land surface physics model, is small compared to the ocean. There are large STD values over the central tropical eastern Pacific due to ENSO variability. The large values over the Gulf Stream and Kuroshio current regions are due to their large interannual and decadal variability of surface temperature [*de Coëtlogon et al*., 2006; *Nakamura et al*., 2012]. Decadal trends in the TOA net flux (1985–2015; Figure 9e) are negative over the central Pacific and positive over the southern ocean and central America. The anomalous positive trend off the west coast of southern Africa is similar to the STD pattern (Figure 9c). The stippling shows where the trend is significant using Mann‐Kendall test at a significance level of 0.05 [*Hipel and McLeod*, 1994]. The *F*_*s*_ trend (Figure 9f) is mostly positive over extratropical oceans but negative over the tropical oceans, particularly over the central eastern Pacific, consistent with *Liu et al*. [2015]. This pattern of change appears to coincide with an increase in cloud amount and albedo in tropical regions and a decrease in midlatitudes detected in the satellite record and simulated by climate models [*Norris et al*., 2016], which has been related to externally forced poleward movement in the large‐scale atmospheric circulation and increasing cloud altitude. Further work connecting secular changes in clouds, circulation, and the energy budget is merited.

The 5 year and longer period multiannual global and hemisphere mean *F*_*T*_ and *F*_*s*_ are shown in Table 2. Hemispheric imbalances in *F*_*T*_ are evident with net energy gain in the Southern Hemisphere and net energy loss in the Northern Hemisphere, except for the anomalous positive 0.24 Wm^−2^ in the Northern Hemisphere during 1995–1999. The large global imbalance during this period was also noted by *Smith et al*. [2015] and coincides with a relatively quiescent period of volcanic activity. Caution in interpreting changes during this period is warranted due to reduced sampling from the ERBS wide field of view instrument which was used to constrain changes in hemispheric mean TOA fluxes. TOA and surface net downward flux in the Southern Hemisphere increase from the 1985–1989 to the 1995–1999 period with relatively stable net flux since 2000.

**Table 2:** Multiannual Mean Net Downward Radiation Fluxes at TOA (*F*_*T*_) and Net Heat Fluxes at Surface (*F*_*s*_)^a^

Period	*F*_*T*_				*F*_*S*_			
	Global	NH	SH	SH‐NH	Global	NH	SH	SH‐NH
1985–1989	0.13	−0.19	0.45	0.64	0.11	−1.44	1.65	3.09
1990–1994	−0.07	−0.39	0.25	0.64	−0.06	−1.66	1.54	3.20
1995–1999	0.73	0.24	1.23	0.99	0.76	−0.90	2.42	3.32
2000–2004	0.59	−0.17	1.35	1.52	0.55	−1.15	2.26	3.41
2005–2009	0.59	−0.17	1.35	1.52	0.58	−0.86	2.03	2.89
2010–2014	0.53	−0.22	1.29	1.51	0.53	−1.24	2.30	3.54
2005–2015	0.59	−0.13	1.31	1.44	0.58	−0.96	2.11	3.07
1985–2015	0.43	−0.13	1.00	1.13	0.42	−1.17	2.02	3.19
2006–2013	0.59	−0.23	1.41	1.64	0.58	−1.11	2.28	3.39

a The units are Wm^−2^. NH: Northern Hemisphere; SH: Southern Hemisphere.

The subtle differences in the way heat is distributed between the Northern and Southern Hemispheres are important in determining global rainfall patterns and climate [*Frierson et al*., 2013; *Loeb et al*., 2016]. Southern Hemisphere minus Northern Hemisphere differences in net downward flux at the TOA approximately double from 0.64 Wm^−2^ for 1985–1994 to 1.5 Wm^−2^ after 2000 while differences are relatively stable at the surface (2.9–3.5 Wm^−2^). The change in TOA fluxes is larger than the uncertainty estimated to be ±0.24 Wm^−2^ related to the homogeneity adjustment applied by *Allan et al*. [2014] and is further supported by agreement with updated estimates of ocean heating rate [*Cheng et al*., 2017].

**Figure 10:**
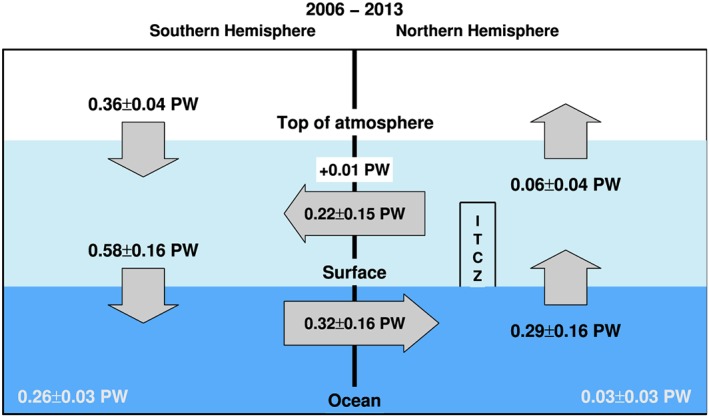
New observations of energy flows in the climate system in petawatts (PW) over 2006–2013 and the location of the Intertropical Convergence Zone (ITCZ). TOA radiation flux is from CERES EBAF anchored to 0.59 W/m^2^ over 2006–2013. Ocean heat storage is based on observations [*Roemmich et al*., 2015].

Using the CERES TOA radiation fluxes anchored to the observed net heat uptake of the Earth system (0.59 Wm^−2^ over 2006–2013 based on *Roemmich et al*. [2015], see section 2), the mass‐corrected and land surface flux‐adjusted ERA‐Interim atmospheric total energy divergences [*Mayer and Haimberger*, 2012; *Liu et al*., 2015] and the reconstructed surface fluxes in this study, the cross‐equatorial heat transports are depicted in Figure 10. This improves estimates by *Loeb et al*. [2016] through additional consideration of the observed heat sinks in the northern and southern oceans [*Roemmich et al*., 2015]. Using CERES TOA data and integrated ERAINT energy divergence and tendency, *Loeb et al*. [2016] estimated net atmospheric energy transport from the Northern Hemisphere to the Southern Hemisphere of 0.24 PW (petawatts) over 2001–2012, and a 0.44 PW net ocean heat transport in the opposite direction, without considering differential heat accumulation in the Northern Hemisphere and Southern Hemisphere oceans. When the observed ocean heat storage is considered (please refer to Table S5 notes for detailed calculations) ocean heat transport is estimated to be 0.31 PW, smaller than *Loeb et al*. [2016]. Using ocean reanalysis and observational data sets and accounting for ocean heating, *Stephens et al*. [2016] estimated the net ocean heat transport of 0.45 PW, remarkably consistent with 0.44 PW of *Loeb et al*. [2016] despite their lack of account for ocean heating differences between hemispheres in *Loeb et al*. [2016]. The 0.9 Wm^−2^ Southern Hemisphere ocean heating calculated by *Stephens et al*. [2016] does not include the deeper ocean heat storage of 0.07 Wm^−2^; including this term decreases the ocean heat transport somewhat (~0.41 PW). Their atmospheric heat transport of 0.33 PW is larger than the 0.24 PW computed by *Loeb et al*. [2016]. It is noticed that both the geodetic weighting and accounting for the number of days per month are considered in calculating the multiannual mean TOA flux using CERES data. Additional analysis suggests that estimates by *Stephens et al*. [2016] may not have accounted for the varying number of days per month which the cross‐equatorial energy flux calculations are sensitive to.

The data period for Figure 10 is from 2006 to 2013. It shows that 0.36 PW is accumulating in the Southern Hemisphere while the Northern Hemisphere is actually losing more energy to space than it is gaining. The uncertainty (0.04 PW) in TOA net flux is from *Johnson et al*. [2016] (0.1 Wm^−2^ ≈ 0.03 PW) and 0.1 Wm^−2^ of possible error between Terra and Aqua (section 3.1). The uncertainty of the atmospheric total hemisphere energy divergence of 0.15 PW is estimated as one standard deviation of the four mass‐corrected divergences from ERAINT, MERRA, JRA55, and JRA55C over 2005–2012. Since the surface flux is the residual of TOA fluxes and atmospheric energy transport, so the uncertainty of *F*_*s*_ is estimated as 0.16 PW using the quadrature addition. After considering the energy required for land/ice heating (see Table S5 notes for details), about 0.58 PW of energy enters the Southern Ocean and 0.29 PW is released from the Northern Ocean. The corresponding net atmospheric energy transport from the Northern Hemisphere to the Southern Hemisphere is 0.22 PW, and the net atmosphere heating is about 0.01 PW. Based on *Roemmich et al*. [2015], the observed heat storage in the Southern Hemisphere ocean is 7.2 × 10^21^ J/yr (~ 0.9 Wm^−2^ or 0.23 PW) for 0–2000 m over 2006–2013 and 0.8 × 10^21^ J/yr (~ 0.1 Wm^−2^ or 0.03 PW) in the Northern Hemisphere ocean. Assuming the deep ocean heat storage of 0.07 Wm^−2^ (~0.03 PW) is in the southern ocean, the overall heat storage in southern ocean is about 0.26 PW. The cross‐equator heat transport by the oceans, primarily the Atlantic, is calculated as 0.32 PW. Much of this transported energy is released into the atmosphere north of the equator (0.29 PW). This extra heating of the Northern Hemisphere atmosphere affects the global atmospheric circulation and may explain why the tropical rain belt (or Intertropical Convergence Zone, ITCZ) is more in the Northern Hemisphere than the south [*Frierson et al*., 2013], which subsequently helps to move a large fraction of the heat back south of the equator by the atmospheric winds (around 0.22 PW).

The estimated hemispheric ocean heat transport (0.32 PW) from our data over 2006–2013 is consistent with *Loeb et al*. [2016] over 2001–2012 when ocean heat storage is accounted for but is much smaller than 0.45 PW estimated by *Stephens et al*. [2016], though this is reduced to 0.41 PW when deeper ocean heat storage is considered and is within the estimated uncertainty. Discrepancies and uncertainties in energy transports are therefore substantial, and further investigations are needed to advance understanding of and to reduce uncertainty in cross‐equatorial energy flows.

## 4 Discussion and Conclusions

An evaluation of global surface energy fluxes computed using top‐of‐atmosphere (TOA) radiation measurements combined with atmospheric energy transports and tendencies from reanalyses is undertaken. The TOA radiation fluxes are updated using estimates of Earth system net heat uptake of 0.59 ± 0.1 Wm^−2^ over 2006–2013, with 0.49 Wm^−2^ by the ocean from 0 to 2000 m [*Roemmich et al*., 2015], 0.07 Wm^−2^ by the deeper ocean [*Loeb et al*., 2012; *Johnson et al*., 2016], and 0.03 Wm^−2^ by melting ice, warming land, and an increasingly warmer and moister atmosphere [*Johnson et al*., 2016]. Following *Allan et al*. [2014], reconstructed estimates are 0.27 ± 0.38 Wm^−2^ for 1985–1999 and 0.59 ± 0.14 Wm^−2^ for 2000–2015 (uncertainty at the 90% confidence level). An increase in energy imbalance of 0.32 Wm^−2^ from the 1985–1999 period to the 2000–2015 period remains likely since the uncertainty relating to the homogeneity between periods is smaller in magnitude at ±0.24 Wm^−2^ [*Allan et al*., 2014]. Regional monthly uncertainty in TOA net radiation is gauged by considering CERES observations and the reconstruction methodology. Evaluation of the surface flux methodology suggests that regional errors in TOA satellite data are small relative to uncertainty related to horizontal energy transports depicted by reanalyses.

Applying the updated radiation budget data, improved estimates of surface energy flux are produced by combination with reanalysis‐based atmospheric energy transports and tendencies. The methodology of *Liu et al*. [2015] is modified using an improved energy budget approach in which unrealistic land surface energy fluxes are adjusted based upon the relation between surface flux and surface temperature change rate at grid points simulated by a high‐resolution climate model including a sophisticated land surface scheme, with additional consideration of the energy required for snowmelt. The modeled and reconstructed surface fluxes display improved agreement (the correlation coefficients for the time series increase from 0.65 to 0.78, see Table S1) compared to *Liu et al*. [2015]. Using this land surface flux constraint, the land surface flux excess or deficit is redistributed to/from the oceans to maintain energy balance, applying a quasi‐normal distribution with the peak at the latitudinal band center, decaying farther away from the center, in order to avoid meridional discontinuity.

The derived turbulent energy flux anomaly variability, calculated from the estimated net surface flux and the CERES EBAF surface radiative flux, shows good agreement with those from the OAFLUX product. The area mean anomaly correlation coefficients ***r***_1_ ≥ 0.60 over most regions defined in Figure 3a. Over the tropical warm pool region where the time series diverge after 2006 (Figure 3e), the composite turbulent energy flux variability from 25 TAO/TRITON/RAMA buoy stations in that region supports our estimation. Over all other selected regions where buoy data are available, the correlations between our estimation and buoy data are all better than those between OAFLUX and buoy data, confirming the improvement of the turbulent flux estimation from our product over the OAFLUX on this aspect. The comparisons of the net surface flux variability between our estimation and those from ocean reanalyses (GODAS, ORAS4, and C‐GLORS05V3) show consistent good agreement over selected regions, with 73% of the correlation coefficients ***r*** ≥ 0.7 and 92% of ***r*** ≥ 0.6. The inferred global meridional ocean heat transports from observed ocean heating and the estimated net surface fluxes using the zonal redistribution method are marginally superior to the ocean transports from other methods based on comparison with observationally based estimates.

The regional and zonal mean surface flux uncertainties are investigated based on the methodology and intercomparison of reanalyses and satellite products. The magnitude of the excess land surface flux can be considered an upper limit on the land surface flux uncertainty based upon the reanalysis energy transports, and monthly regional differences are up to 40 Wm^−2^ in magnitude while zonal monthly mean differences can be around 25 Wm^−2^. The redistributed monthly mean flux over oceans can also reach 25 Wm^−2^ around 55°N due to the relatively small ocean area over that latitude band.

From the multiannual mean (2001–2005) divergence differences over land before and after the adjustment, the zonal mean shows differences of between 4 and 10 Wm^−2^ north of 55°N and around 14 Wm^−2^ over the tropics. The corresponding change over the oceans peaks at ~10 Wm^−2^ around 55°N and becomes quite small over the remaining oceans due to relatively large ocean areas and the smoothing method used in the redistribution. It is conceivable that regional uncertainties comparable with the discrepancies up to 40 Wm^−2^ over land also apply over the ocean. However, complex coastline and terrain are likely to increase errors in energy transport calculations over land and coastal regions, and the adjustment toward physically consistent energy fluxes is employed to reduce actual errors. Therefore, such biases can be considered as an upper limit for regional monthly mean surface flux uncertainty.

The discrepancy of the global surface fluxes is large from five atmospheric reanalysis data sets, and the zonal mean standard deviation is about 11 Wm^−2^. After using the mass‐corrected divergences and a common TOA radiation input, the discrepancies are greatly reduced and the zonal mean standard deviation is about 3.0 Wm^−2^. The standard deviation of the multiannual (2001–2008) zonal means over oceans are generally 1–5 Wm^−2^ except for near the North Pole.

The surface energy flux generated in the present study does not account for energy used in melting ice. Based on *Stocker et al*. [2013], Greenland is losing mass at ~200 Gt/yr (= 2 × 10^14^ kg/yr). Using latent heat of water 3.34 × 10^5^ J/kg and the area of Greenland ~2.2 × 10^12^ m^2^, the estimated energy flux over Greenland needed for ice melt will be about 1 Wm^−2^. This is significant locally but is small compared with the uncertainties from our constraint procedures and the spread of the reanalyses. The melting of sea ice requires energy as well, but we assume the magnitude to be much smaller [*Hansen et al*., 2011].

Applying the updated TOA and surface energy flux estimates, the cross equator transports are computed. There is a positive TOA radiative imbalance of 0.36 PW in the Southern Hemisphere while the Northern Hemisphere is losing more energy to space than it is gaining, consistent with *Loeb et al*. [2016] and *Stephens et al*. [2016]. After considering the heat storage of 0.26 PW in the Southern Ocean [*Roemmich et al*., 2015], the cross‐equator heat transport by the oceans is estimated as 0.32 ± 0.16 PW over 2006–2013, which is consistent with *Loeb et al*. [2016] over 2001–2012 when ocean heat storage is accounted for, but is much smaller than 0.45 PW estimated by *Stephens et al*. [2016], though this is reduced to 0.41 PW when deeper ocean heat storage is considered. Discrepancies using different approaches indicating that more investigation is needed. Based upon our calculations, 0.22 ± 0.15 PW of heat is transported back south across the equator by the atmospheric winds.

Combined satellite‐/reanalysis‐based estimates of net surface flux data have been widely used by other investigators [*Trenberth et al*., 2001, 2009; *Loeb et al*., 2016; *Williams et al*., 2015; *Valdivieso et al*., 2015; *Senior et al*., 2016; *Roberts et al*., 2017; *Mayer et al*., 2016; *Kato et al*., 2016; *Trenberth and Fasullo*, 2017] for surface flux comparisons and model simulation validations; further evaluation of uncertainty including comparison with buoy data will help to improve the utility of these products in understanding and monitoring the climate system. Given the large uncertainties of the observations and the limit of our zonal redistribution approach, other methodologies, such as the inverse model method which has been used in oceanography for many years [*Lumpkin and Speer*, 2007], could be applied. Improved estimates of calculated horizontal energy transports would require characterization of uncertainty in the determining state variables such as temperature, water vapor, and wind. Applying inverse models and making energy flux adjustments at each time step to ensure physical consistency is necessary to improve over the methods currently applied.

## Supporting information



Supporting Information S1
