# Supplementary material for: Evaluation of satellite and reanalysis‐based global net surface energy flux and uncertainty estimates
Source: J Geophys Res Atmos. 2017 Jun 26;122(12):6250–72. doi: 10.1002/2017JD026616 (PMC5530441; doi:10.1002/2017JD026616)
Supplement: Supplementary file 1 — Supporting Information S1 [file JGRD-122-6250-s001.pdf]

SUPPLEMENTARY INFORMATION

Evaluation of satellite and reanalysis-based global net surface energy flux and uncertainty estimates

Chunlei Liu[1,2], Richard P. Allan[1,2,3], Michael Mayer[4], Patrick Hyder[5], Norman G. Loeb[6], Chris D. Roberts[5], Maria Valdivieso[1], John M. Edwards[5] and Pier-Luigi Vidale[1,3]

[1] *Department of Meteorology, University of Reading, Reading, UK*

[2] *National Centre for Earth Observation, UK*

[3] *National Centre for Atmospheric Science, UK*

[4] *Department of Meteorology and Geophysics, University of Vienna, Austria.*

[5] *Met Office, Exeter, UK*

[6] *NASA Langley Research Centre, Hampton, Virginia, USA*

Table S1. The correlation coefficients (***r***) between the modelled and reconstructed surface flux anomalies, the standard deviation (STD) ratio of six month anomaly running means, and the mean effective heat capacity ***C*** from five UPSCALE members (xgxqe – xgxqi). The ***r*** and STD from global mean relations are in brackets. The ***C*** is the 12 month mean ± one standard deviation.

Data set	***r***	STD ratio	***C (W/m²/(K/day))***
xgxqe	0.78 (0.65)	2.14 (2.62)	29.3±2.88
xgxqf	0.77 (0.68)	1.81 (2.35)	29.0±2.63
xgxqg	0.77 (0.61)	2.09 (2.05)	28.8±2.51
xgxqh	0.78 (0.64)	1.84 (2.32)	28.9±2.67
xgxqi	0.74 (0.65)	1.82 (2.80)	29.1±2.72

Table S2. Selected regions and correlations between data sets. r_1 and r_2 are anomaly correlation coefficients between our estimated turbulences (from F_S with and without land flux adjustment) and those from OAFLUX. r_3 and r_4 are the corresponding correlation coefficients with composite buoy data. r_5 is the anomaly correlation coefficient between OAFLUX and the buoy data. The reference period is from 2001-2005.

Region	Coverage	r_1	r_2	r_3	r_4	r_5	Number of buoy station
Global ocean		0.68	0.55				
A	45–55 °N, 150–220 °E	0.85	0.88				
B	16–26 °N, 180–250 °E	0.69	0.70				
C	8 °S–10 °N, 80–160 °E	0.39	0.33	0.51	0.52	0.04	25
D	2–10 °N, 190–280 °E	0.24	0.29	0.34	0.28	0.20	18
E	8 °S–1 °N, 190–260 °E	0.73	0.74	0.19	0.18	0.16	19
F	15 –30 °S, 60–110 °E	0.74	0.75				
G	25 – 45 °S, 200–250 °E	0.61	0.59				
H	45 – 65 °N, 290–360 °E	0.81	0.81				
I	6–25 °N, 290–340 °E	0.75	0.72	0.14	0.14	0.08	6
J	5 °S –5 °N, 320–355 °E	0.24	0.25	0.32	0.32	0.20	8
K	6 –25 °S, 320–360 °E	0.69	0.69	0.22	0.22	0.05	4

Regions D and J (Fig. 3f,l) are in the tropical areas of Pacific and Atlantic, respectively. The correlations of 0.34 and 0.32 between our estimations and the buoy data are better than those (~0.24) between our estimation and the OAFLUX data, while the correlations between OAFLUX and buoy data over these two regions are slightly lower. Over regions E, I and K (Fig. 3g,k,m), the correlations (0.19, 0.14 and 0.22) between our estimations and the buoy data are much lower than those (0.73, 0.75 and 0.69) between our estimation and OAFLUX data, but the correlations between OAFLUX and buoy data are even lower (0.16, 0.08 and 0.05).

Table S3. Anomaly correlation coefficients of the net surface fluxes between different data sets over the global ocean and selected regions (A-K) as shown in Fig. 3a.

	Between Derived and			Between C-GLORS05V3 and		Between GODAS and
	C-GLORS05V3	GODAS	ORAS4	GODAS	ORAS4	ORAS4
Global ocean	0.29	0.13	0.55	-0.29	0.29	0.46
A	0.82	0.67	0.86	0.70	0.82	0.82
B	0.78	0.76	0.83	0.64	0.80	0.82
C	0.78	0.73	0.82	0.71	0.87	0.84
D	0.81	0.58	0.82	0.50	0.94	0.61
E	0.87	0.70	0.86	0.80	0.96	0.85
F	0.34	0.68	0.80	0.69	0.75	0.84
G	0.66	0.83	0.83	0.62	0.79	0.91
H	0.91	0.77	0.69	0.79	0.63	0.70
I	0.84	0.79	0.87	0.72	0.90	0.82
J	0.61	0.48	0.51	0.62	0.89	0.55
K	0.75	0.64	0.78	0.66	0.79	0.78

Table S4. Mean biases and standard deviations (in brackets) of the monthly meridional transport differences between the reconstruction and observations. The final column is an average over all monthly differences. Units are in PW.

		Ganachaud and Wunsch (2003)	Talley (2003)	Lumpkin and Speer (2007)	Macdonald (1988)	Bryden and Imawaki (2001)	Johns et al. (2011)	All data
Global ocean	No Adjustment	-0.28 (0.20)	-0.43 (0.32)	-0.38 (0.29)				-0.37 (0.26)
	Whole ocean	-0.16 (0.15)	-0.34 (0.34)	-0.23 (0.18)				-0.25 (0.24)
	SH and NH oceans	-0.07 (0.13)	-0.25 (0.32)	-0.18 (0.19)				-0.17 (0.23)
	Zonally weighted	-0.06 (0.18)	-0.17 (0.25)	-0.14 (0.27)				-0.12 (0.22)
Atlantic	No Adjustment	-0.20 (0.16)	0.05 (0.21)	-0.10 (0.12)	-0.11 (0.18)	-0.02 (0.18)	-0.32	-0.08 (0.19)
	Whole ocean	-0.18 (0.16)	0.06 (0.22)	-0.09 (0.11)	-0.10 (0.18)	0.00 (0.18)	-0.31	-0.06 (0.19)
	SH and NH oceans	-0.13 (0.15)	0.10 (0.24)	-0.05 (0.09)	-0.05 (0.18)	0.05 (0.17)	-0.27	-0.02 (0.19)
	Zonally weighted	-0.06 (0.15)	0.18 (0.22)	0.02 (0.09)	-0.03 (0.17)	0.12 (0.16)	-0.16	0.05 (0.18)

Table S5. The energy flow at TOA and surface, including energy storage in the atmosphere and ocean, as well as the land/ice heating over 2006-2013. Bold values in brackets are used in Fig. 7. All units are in PW.

	Global	SH	NH
F_T	0.3009	0.3600 (**0.36**)	-0.0591 (**-0.06**)
Atmosphere convergence	0.0029 (**0.01**)	-0.2222	0.2251
Transport NH→SH		0.2237 (**0.22**)	
F_s	0.2980	0.5822 (**0.58**)	-0.2842 (**-0.21**)
Land/Ice heating	0.0142	0.0037	0.0087
Energy entering the ocean		0.5785 (**0.58**)	0.2929 (**0.29**)
Ocean heat storage		0.2639 (**0.26**)	0.0254 (**0.03**)
Transport SH→NH		0.3145 (**0.32**)	

The calculation details are as follows:
(1) Based on data in table 2 over 2006-2013, the total atmosphere heating
H(air) = 0.3009 - 0.2980 = 0.0029PW **(~ 0.01**PW, also see Fig. 10).
The atmosphere energy convergence (DIV)
DIV(SH) = 0.3600 - 0.5822 = -0.2222PW
DIV(NH) = -0.0591 – (-0.2842) = 0.2251PW
The hemisphere atmosphere heat transport AHT = (0.2251+0.2222 - 0.0029)/2 = 0.2222PW (~**0.22**PW)

(2) The net downward surface flux Fs = 0.2980PW, the energy heating land/ice is about 0.03×0.255×2 (Johnson et al. 2016) - 0.0029 (atmosphere) = 0.0124PW. Assume 30% of land/ice heating happens in SH, then the heat entering the ocean is
F(SH) = 0.5822 - 0.0124×30% = 0.5785PW (~**0.58**PW)
F(NH) = 0.2842 + 0.0124×70% = 0.2929PW (~**0.29**PW, released from the ocean)

(3) Based on the observed hemisphere heat storage in the ocean (*Roemmich et al.* 2015, 0-2000m),
S(SH) = $7.2\times10^{21}/(2.55\times10^{14}\times365\times24\times3600)$ = 0.8953 Wm^{-2} = 0.2283PW
S(NH) = $0.8\times10^{21}/(2.55\times10^{14}\times365\times24\times3600)$ = 0.0995 Wm^{-2} = 0.0254PW (**0.03**PW)
Assume deep ocean heat storage of 0.07 Wm^{-2} (~0.0357PW) is all in SH ocean, then
S(SH) = 0.2283 + 0.0357 = 0.264PW (~**0.26**PW)

(4) The ocean heat transport (OHT) in the ocean is
0.5785 - 0.264 = 0.3145PW (~**0.32**PW)
The estimated flux released from the NH ocean is
0.3145-0.0254 = 0.2891PW (~**0.29**PW). This is consistent with the value in step(2).

(5) The uncertainty of ±0.04PW at TOA is the quadrature addition from ±0.1 Wm^{-2} of Johnson et al. [2016] and ±0.1 Wm^{-2} of possible equipment error between Terra and Aqua. The uncertainties 0.15PW of atmosphere divergence are based on 4 mass corrected atmospheric divergences from ERAINT, MERRA, JRA55, JRA55C over 2005-2012. It is the standard deviation of 4 multiannual means. The surface flux uncertaint 0.16PW is from quadrature addition of 0.04PW and 0.15PW. Heat storage error is based on observation of *Roemmich et al.* [2015].

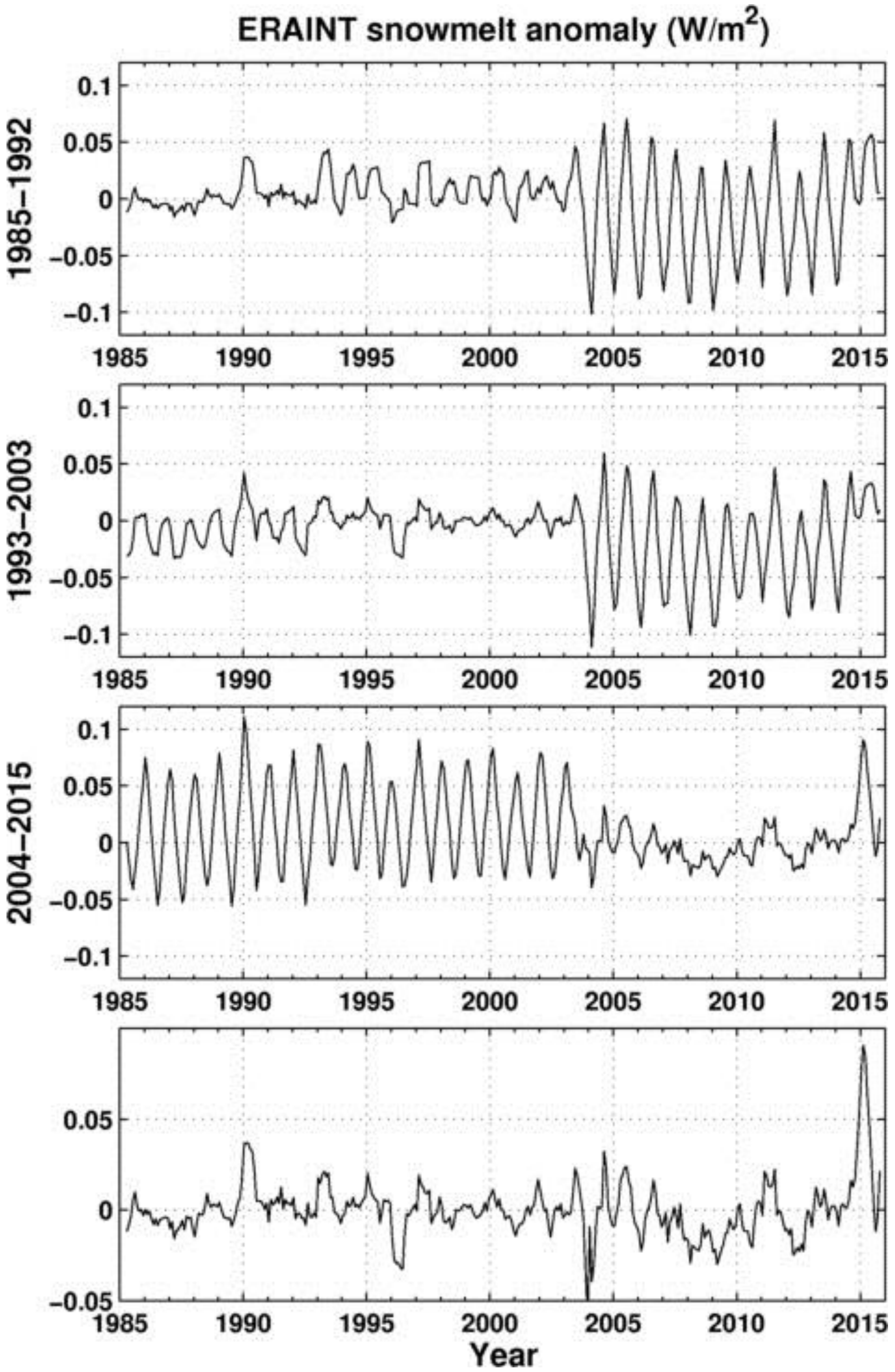

Fig. S1. Global land area mean snowmelt anomaly from ERAINT. Reference period is (a) 1985-1992; (b) 1993-2003 and (c) 2004-2015; (d) is the joint anomalies from three time periods. All lines are 6 month running means.

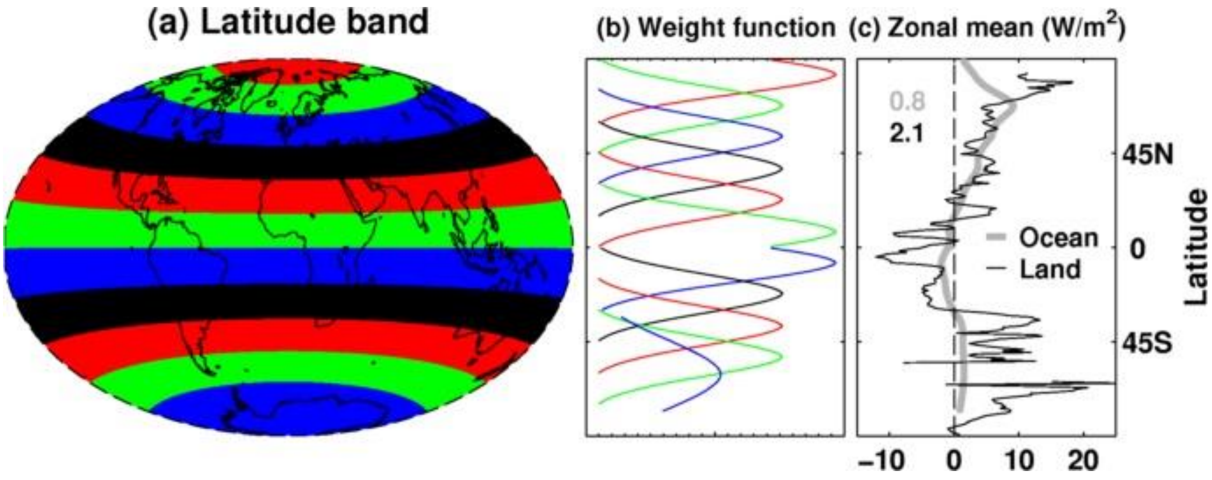

Fig. S2. (a) and (b) are schematic diagrams illustrating the eleven latitudinal bands and the corresponding weight curves. Note the corresponding band and weight curve are in the same colour. The weight curves on both sides of the Equator and near the Poles are not symmetrical. (c) is the zonal mean of the net surface energy flux deficit (positive) over land and the redistribution over the oceans, the corresponding multiannual means (2006-2013) over the global land and the global ocean are also displayed.

The multiannual global mean (2006-2013) land surface flux deficit (positive here) is about 2.1 Wm^{-2} and

- for case (ii) in section 3.3, the mean redistribution over the global ocean is ~0.8 Wm^{-2}.
- for case (iii), the redistributions are 2.0 Wm^{-2} and -0.03 Wm^{-2} over the northern and southern hemisphere oceans, respectively.
- for case (iv), the redistribution over oceans is latitude dependent as shown in Fig. S2c as a thick grey line.

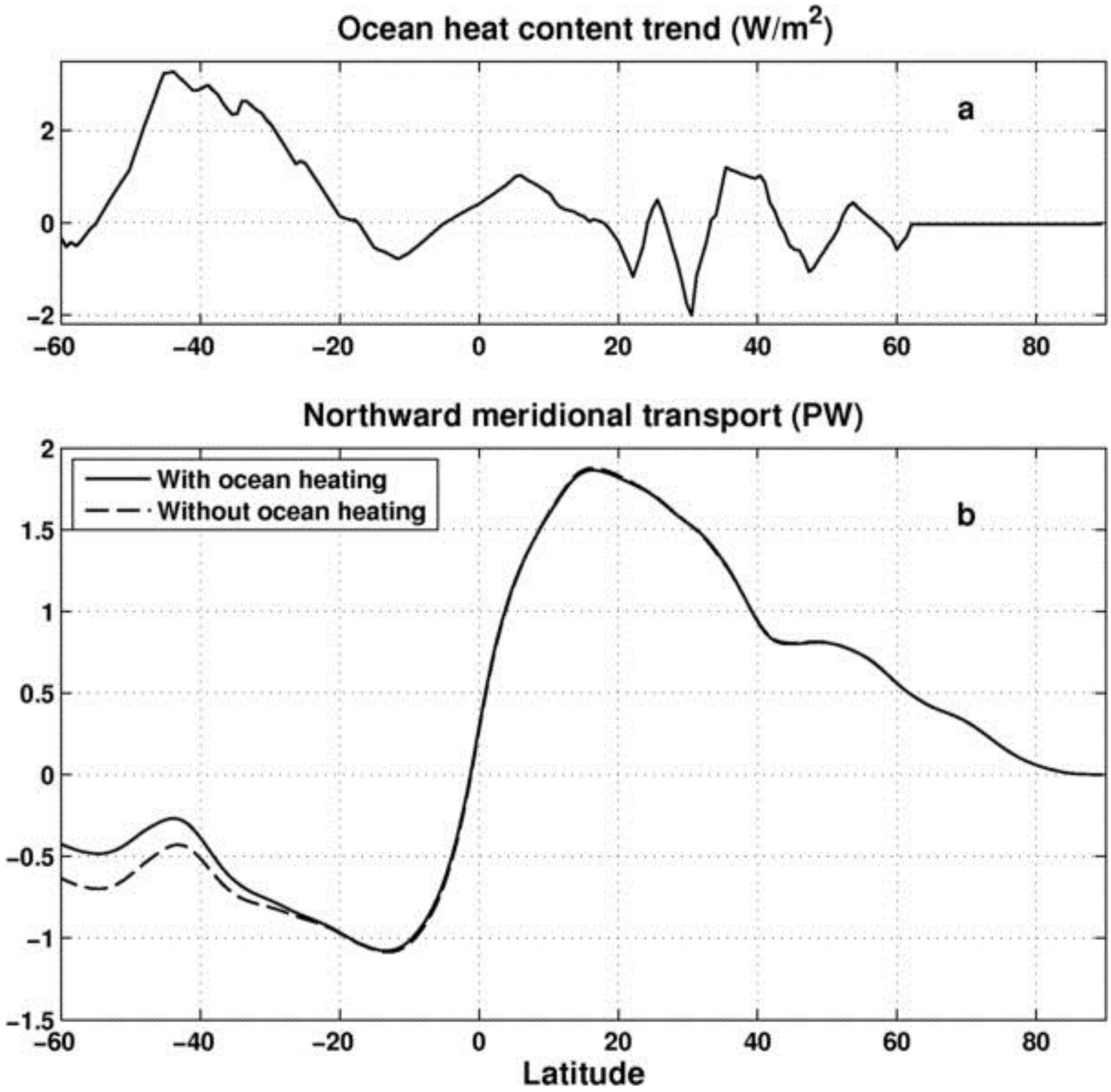

Fig. S3. (a) Estimated zonal ocean heat content trend (W/m^2) from *Roemmich et al.* [2015] for 0-2000m; (b) inferred northward meridional transports (PW) with and without ocean heatings.

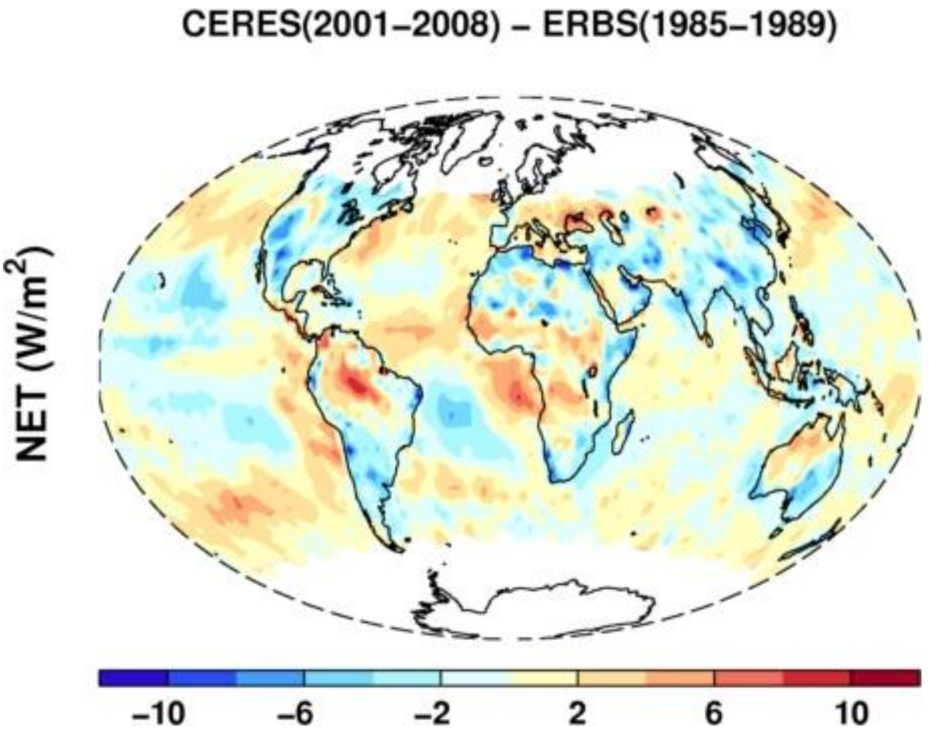

Fig. S4. Multiannual mean difference of TOA net radiation fluxes between CERES and ERBS.
